# PROTAC-mediated conditional degradation of the WRN helicase as a potential strategy for selective killing of cancer cells with microsatellite instability

**DOI:** 10.1038/s41598-024-71160-5

**Published:** 2024-09-06

**Authors:** Vikram Tejwani, Thomas Carroll, Thomas Macartney, Susanne Bandau, Constance Alabert, Giulia Saredi, Rachel Toth, John Rouse

**Affiliations:** 1 MRC Protein Phosphorylation and Ubiquitylation Unit, University of Dundee, Dundee, DD1 5EH UK; 2 Division of Molecular, Cell and Developmental Biology, School of Life Sciences, Wellcome Trust Biocentre, University of Dundee, Dundee, DD1 5EH UK

**Keywords:** WRN, Werner syndrome, MSI, Microsatellite instability, Cancer, PROTAC, Degrader, DNA, Targeted therapies, DNA damage response, DNA mismatch repair, DNA repair enzymes

## Abstract

Multiple studies have demonstrated that cancer cells with microsatellite instability (MSI) are intolerant to loss of the Werner syndrome helicase (WRN), whereas microsatellite-stable (MSS) cancer cells are not. Therefore, WRN represents a promising new synthetic lethal target for developing drugs to treat cancers with MSI. Given the uncertainty of how effective inhibitors of WRN activity will prove in clinical trials, and the likelihood of tumours developing resistance to WRN inhibitors, alternative strategies for impeding WRN function are needed. Proteolysis-targeting chimeras (PROTACs) are heterobifunctional small molecules that target specific proteins for degradation. Here, we engineered the *WRN* locus so that the gene product is fused to a bromodomain (Bd)-tag, enabling conditional WRN degradation with the AGB-1 PROTAC specific for the Bd-tag. Our data revealed that WRN degradation is highly toxic in MSI but not MSS cell lines. In MSI cells, WRN degradation caused G_2_/M arrest, chromosome breakage and ATM kinase activation. We also describe a multi-colour cell-based platform for facile testing of selective toxicity in MSI versus MSS cell lines. Together, our data show that a degrader approach is a potentially powerful way of targeting WRN in MSI cancers and paves the way for the development of WRN-specific PROTAC compounds.

## Introduction

To prevent the potentially genome-destabilizing effects of DNA damage, cells are equipped with a range of DNA damage repair (DDR) pathways. The DNA mismatch repair (MMR) pathway, for example, detects and repairs mismatches introduced during genome replication^1,2^. Efficient MMR requires the products of several key genes including *MLH1*, *PMS2*, *MSH2* and *MSH6*^3^. MSH2/6 dimers bind to mismatched base errors, while MLH1-PMS2 direct the excision step and fill-in synthesis^4,5^. Germline mutations in MMR genes cause diseases such as Lynch syndrome, which is associated with a predisposition to multiple cancers including colon and endometrial cancers^6,7^. In regions of the genome containing microsatellites—short tracts of repetitive sequences, inappropriate primer-template annealing and/or polymerase slippage can lead to insertion/deletions loops (IDLs) which can be repaired by MMR^8,9^. Microsatellites become unstable in MMR-defective cells, in the form of expansions that can be detected in PCR-based assays^10^. Microsatellite instability (MSI) has been observed in approximately 15% of all colorectal cancers, and in endometrial, ovarian and gastric cancers^11,12^. It has also been estimated that 22% of all western gastric cancers are MSI + . While it is not well understood whether genetic or environmental factors drive this phenomenon, gastric cancers occur at particularly high frequencies in South Korea (42 per 100K age-standardized rate), Mongolia (32 per 100K age-standardized rate), Japan (30 per 100K age-standardized rate), and China (23 per 100K age-standardized rate)^13,14^.

There are currently no therapies for MSI cancers that directly target deficiencies in the MMR system^15,16^. There is evidence however, that MSI tumours are amenable to immune checkpoint blockade^17–20^. For example, combinatorial blockage of cytotoxic T lymphocyte associated protein 4 (CTLA-4) and programmed cell death protein 1 (PD-1) checkpoint receptors led to increased progression-free survival (PFS) and overall survival (OS) rates in MSI colorectal cancer patients^19,20^. While it is not exactly clear why MSI tumours are more responsive to immune checkpoint blockade, increased neoantigen production in cells with MMR defects may potentiate this vulnerability^17,18^. Although MSI tumours are amenable to immune checkpoint blockade, there are examples of MSI colorectal cancer patients being refractory or having intrinsic resistance^18,19,21^. Furthermore, toxicity associated with immune checkpoint blockade agents in MSI patients is a major limitation^22^. Therefore, there is a need for novel therapies that exploit the vulnerabilities of MSI cancers.

Several independent studies recently identified a member of the RecQ family of helicases, the Werner syndrome helicase (WRN), as a “synthetic lethal” (SL) target in MSI cancer cells^15,16,23,24^. Depletion of WRN in MSI cells led to an increase in DNA double strand breaks (DSBs), alterations in cell cycle progression and a decrease in overall cell viability and proliferation compared with WRN-depleted microsatellite-stable (MSS) cells^15,16,24^. While it is not explicitly clear why MSI cells are so critically dependent on WRN for survival, recent studies suggest a link to the role of WRN at (TA)_n_ dinucleotide repeats. These repeat sequences can self-anneal to form DNA cruciform structures, which are efficiently detected and removed by MMR^25–27^. In cells with MMR defects however, these structures accumulate and WRN activity becomes vital for resolving them^25,26^. When WRN is depleted from MSI cells, these cruciform structures are thought to persist into mitosis and are eventually cleaved by the SLX4 complex, leading to DNA breaks in regions of the genome that contain (TA)_n_ repeats^25–27^. Furthermore, inhibiting WRN activity results in the helicase being trapped on chromatin and subsequently targeted for proteasomal degradation via the p97/VCP axis only in MSI but not MSS cell lines^28^.

Taken together, the findings described above point to WRN helicase as an exciting drug target for the treatment of MSI + cancers. The development of potent and specific WRN helicase inhibitors for cancer treatment is challenging in principle, not least because of the high level of similarity between the helicase domains of other RecQ family members^29–31^. There have been attempts made to generate translational small molecule inhibitors of WRN in the past like NSC19630^32^, ML216^33^ and NSC617145^34^. However, these molecules have failed to progress to clinical studies due to selectivity and potency issues^31^. More recently, high-throughput screens have yielded non-covalent^35^ and covalent^36^ small-molecule WRN inhibitors. Currently, there are at least two compounds, VVD-214^37^ and HRO761^38^ that are in early-stage clinical trials for WRN inhibition in MSI + cancers, and it remains to be seen how effective and well tolerated they are in patient populations.

**Fig. 1:**
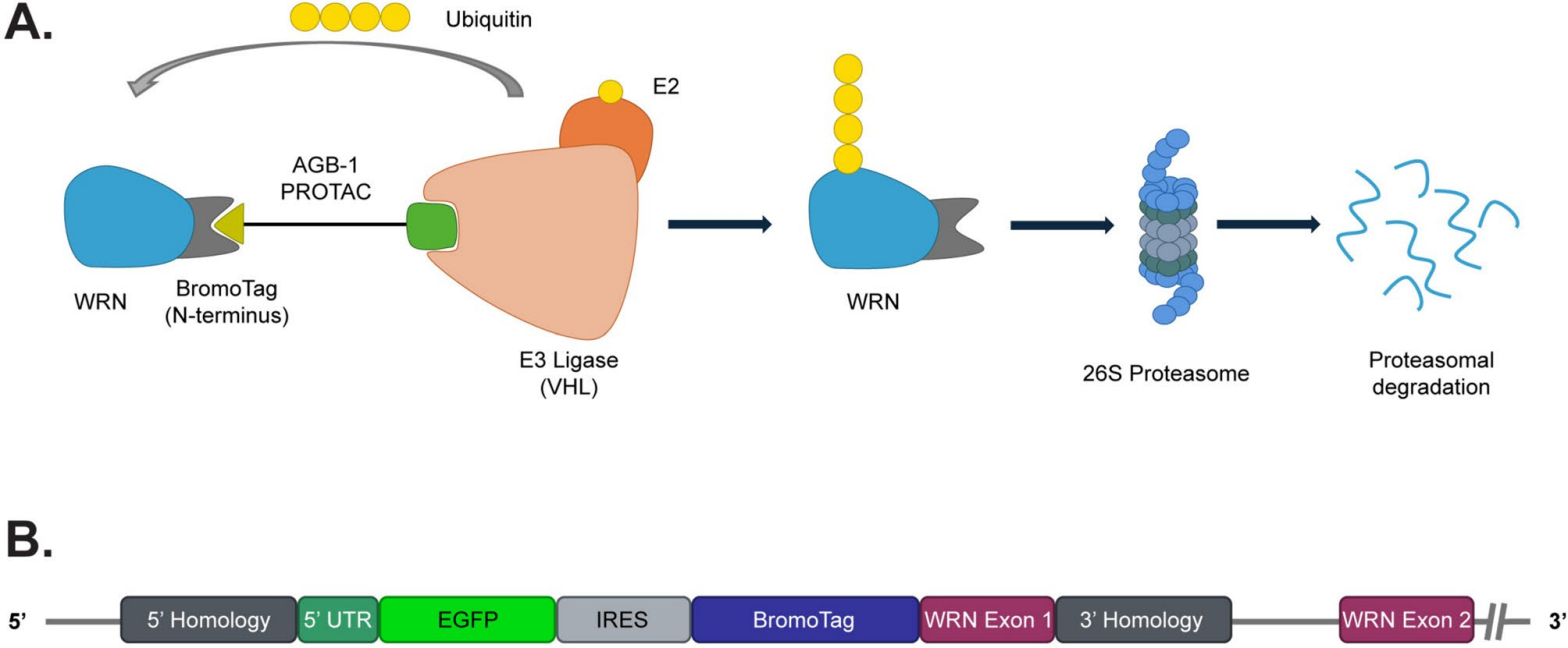
Using the Bd-Tag system to achieve conditional WRN degradation. (**A**) Schematic representing the mechanism of Bd-WRN degradation, upon addition of the AGB-1 PROTAC, via the 26S proteasome. (**B**) Representation of the WRN locus after CRISPR Cas/9 mediated knock-in of the Bd-Tag construct, comprised of EGFP for the purposes of selecting single cell clones where the construct has integrated in the genome, an IRES site and the Bd-Tag cassette, on the N-terminus of the WRN gene product.

Proteolysis-targeting chimeras (PROTACs) offer several distinct advantages over activity-based inhibitors^39,40^. PROTACS are heterobifunctional molecules comprised of a ligand for the protein of interest connected via a linker to an E3 ubiquitin ligase-recruiting ligand (Fig. 1A)—the VHL or CRBN substrate-targeting subunits of CRL-type E3 ligases being the most prevalent^41,42^. The degradation of the target protein is induced by the formation of a ternary complex between the PROTAC, the E3, and the target, resulting in target ubiquitination and degradation by the 26S proteasome. This can result in rapid and persistent depletion of the target protein^42,43^.

Here, we describe a study wherein we engineered the *WRN* locus in MSI and MSS cell lines so that the WRN gene product is fused with a Bromo-domain (Bd)-tag. This is recognized by the AGB-1 PROTAC which recruits Bd-tagged proteins to the VHL subunit of the cullin RING ligase (CRL) CUL2^VHL^ (Fig. 1A)^44^. Tagging WRN in this manner enabled rapid time- and dose-dependent WRN degradation upon addition of AGB-1 to cells, in a manner that was dependent on the 26S proteasome. WRN degradation was cytotoxic in MSI but not MSS cells, and was accompanied by DNA breakage, checkpoint activation and increased nuclear size. We also describe the development of a quantitative multicolour competition assay (MCA) for testing candidate compounds for preferential killing of MSI versus MSS cells. Our findings serve as a proof-of-concept that a PROTAC approach could be a suitable alternative to WRN inhibition, addressing an unmet clinical need for the treatment of MSI cancers.

## Results

### Conditional degradation of WRN in MSI and MSS cells

**Fig. 2:**
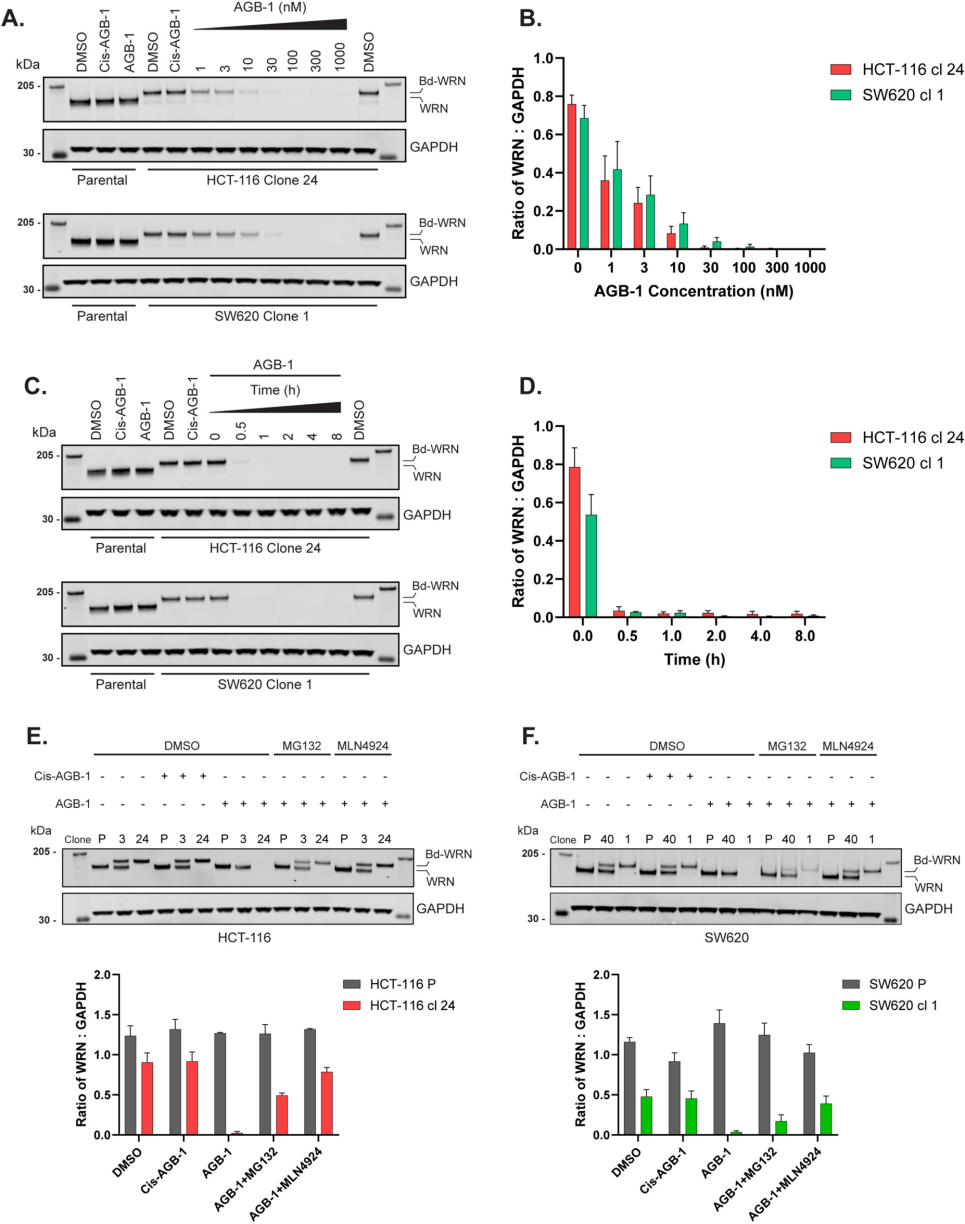
Rapid, PROTAC-inducible and proteasome dependent WRN degradation in MSI HCT-116 clone 24 and MSS SW620 clone 1. (**A**) Representative SDS-PAGE and western blot analysis of lysates from HCT-116 clone 24 (top) and SW620 clone 1 (bottom) showing degradation of Bd-WRN with increasing concentrations of AGB-1, after 3 h of treatment. 3 h Cis-AGB-1 (1 μM) and DMSO (0.1%) treatments were used as controls. Blots from one of three biological repeats are shown. (**B**) Densitometric quantification of WRN normalised to the loading control, GAPDH in response to AGB-1 dose response in (**A**) for HCT-116 clone 24 (red) and SW620 clone 1 (green). Data are represented as mean ± SEM from three biological repeats (*n* = 3). (**C**) Western blot analysis showing the time-dependent degradation of Bd-WRN in the same clones as in (**A**), at a fixed AGB-1 concentration (0.3 μM). Parental cells were also included for each cell type. 8 h Cis-AGB-1 (1 μM) and DMSO (0.1%) treatments were used as controls. Blots from one of three biological repeats are shown. (**D**) Densitometric quantification of WRN normalised to the loading control, GAPDH in response to AGB-1 time course in (**C**) for HCT-116 clone 24 (red) and SW620 clone 1 (green). Data are represented as mean ± SEM from three biological repeats (*n* = 3). (**E**) Western blot analysis showing that AGB-1 mediated degradation of Bd-WRN is dependent on the proteasome in HCT-116 parental cells (P), clone 3 (untagged and Bd-tagged WRN) and clone 24 (Bd-tagged WRN only). Cells were preincubated for 1 h with the 26S proteasome inhibitor MG132 (50 μM) or the NEDDylation inhibitor MLN4924 (3 μM) or DMSO (0.1%) before treatment with 0.3 μM AGB-1 or Cis-AGB-1 for a further 3 h. Blots from one of three biological repeats are shown. Quantification (mean ± SEM) of WRN: GAPDH intensity from three biological repeats (*n* = 3) is shown in the panel below for HCT-116 P (gray) and HCT-116 clone 24 (red). (**F**) The same as in (**E**) done with SW620 parental cells (P), clone 40 (untagged and Bd-tagged WRN) and clone 1 (Bd-tagged WRN only). Quantification (mean ± SEM) of WRN: GAPDH intensity from three biological repeats (*n* = 3) is shown in the panel below for SW620 P (gray) and SW620 clone 1 (green).

We used CRISPR/Cas9 to modify the *WRN* locus to add an inducible degron to the N-terminus of the WRN gene product. The Bd-tag is a modified Bromo-domain from Brd4 that binds to the small molecule proteolysis targeting chimera (PROTAC) AGB-1. This in turn, recruits the E3 ligase, VHL, to enable target degradation, in this case, WRN (Fig. 1A)^44^. Two MSS (SW620 and Caov-3) and two MSI (HCT-116 and SW48) cell lines were transfected with two plasmids encoding the WRN-specific sense and antisense guide (g) RNA pair, the Cas9 D10A nickase^45,46^ and a dsDNA donor construct bearing an EGFP tag for single cell isolation, an IRES and Bd-tag, flanked by homology arms to direct integration of the cassette at the *WRN* locus (Fig. 1B). After selecting GFP + cells by FACS, single cell clones were tested for AGB-1-dependent WRN degradation and candidate positive knock-in (KI) clones were verified using PCR analysis (Fig. S1A–F; see “Materials and methods”). Candidate Bd-WRN clones were then selected for further validation. As shown in Fig. 2A, Bd-WRN in HCT-116 clone 24 showed a 15 kDa size shift compared with the untagged form of WRN seen in parental cells. Exposure of HCT-116 clone 24 to increasing concentrations of AGB-1 induced a dramatic decrease in Bd-WRN band intensity, with maximal degradation observed at 100 nM (Fig. 2A, B). Similar dose response results were obtained for Bd-WRN in SW620 clone 1 and other HCT-116, SW620, Caov-3 and SW48 Bd-WRN cell lines (Figs. 2A, B, S2A, B and S3A). Treatment of cells with Cis-AGB-1, a derivative of AGB-1 containing *cis*- instead of *trans*-hydroxyproline which abrogates binding to VHL^44^, had no apparent effect on Bd-WRN band intensity. Time course experiments revealed rapid, time-dependent Bd-WRN degradation in all four cell lines, with maximal degradation evident between 1 and 2 h of AGB-1 exposure. Again, Cis-AGB-1 had no effect on Bd-WRN intensity even after 8 h and untagged WRN was not affected after treating parental cell lines with AGB-1 (Figs. 2A, C, S2A-D and S3A, B). Similar time course results were obtained for Bd-WRN in SW620 clone 1, and other HCT-116, SW620, Caov-3 and SW48 Bd-WRN cell lines (Figs. 2C, D, S2C, D and S3B).

AGB-1 recruits Bd-tagged target proteins to the cullin-based E3 ligase CUL2^VHL^ which ubiquitylates target proteins for proteasomal degradation^44^. We next tested the dependence of Bd-WRN degradation on the 26S proteasome and on NEDDylation, a post-translational modification required for cullin activity^47,48^. As shown in Figs. 2E and S2E, degradation of Bd-WRN after AGB-1 exposure to HCT-116 clone 24 is blocked by the 26S proteasome inhibitor MG132 and by the NEDDylation inhibitor MLN4924. We also tested HCT-116 clone 3, which has both untagged and Bd-tagged WRN, revealing MG132 and MLN4924-sensitive degradation of Bd-WRN without affecting the untagged allele (Figs. 2E and S2E). Similar results were obtained with SW620 clone 1, and other HCT-116, SW620, Caov-3 and SW48 Bd-WRN cell lines (Figs. 2F, S2F and S4A–F). Thus, AGB-1 enables rapid, conditional, proteasome-dependent degradation of Bd-WRN in cells.

### Conditional WRN degradation is cytotoxic in MSI cells but not in MSS cells

**Fig. 3:**
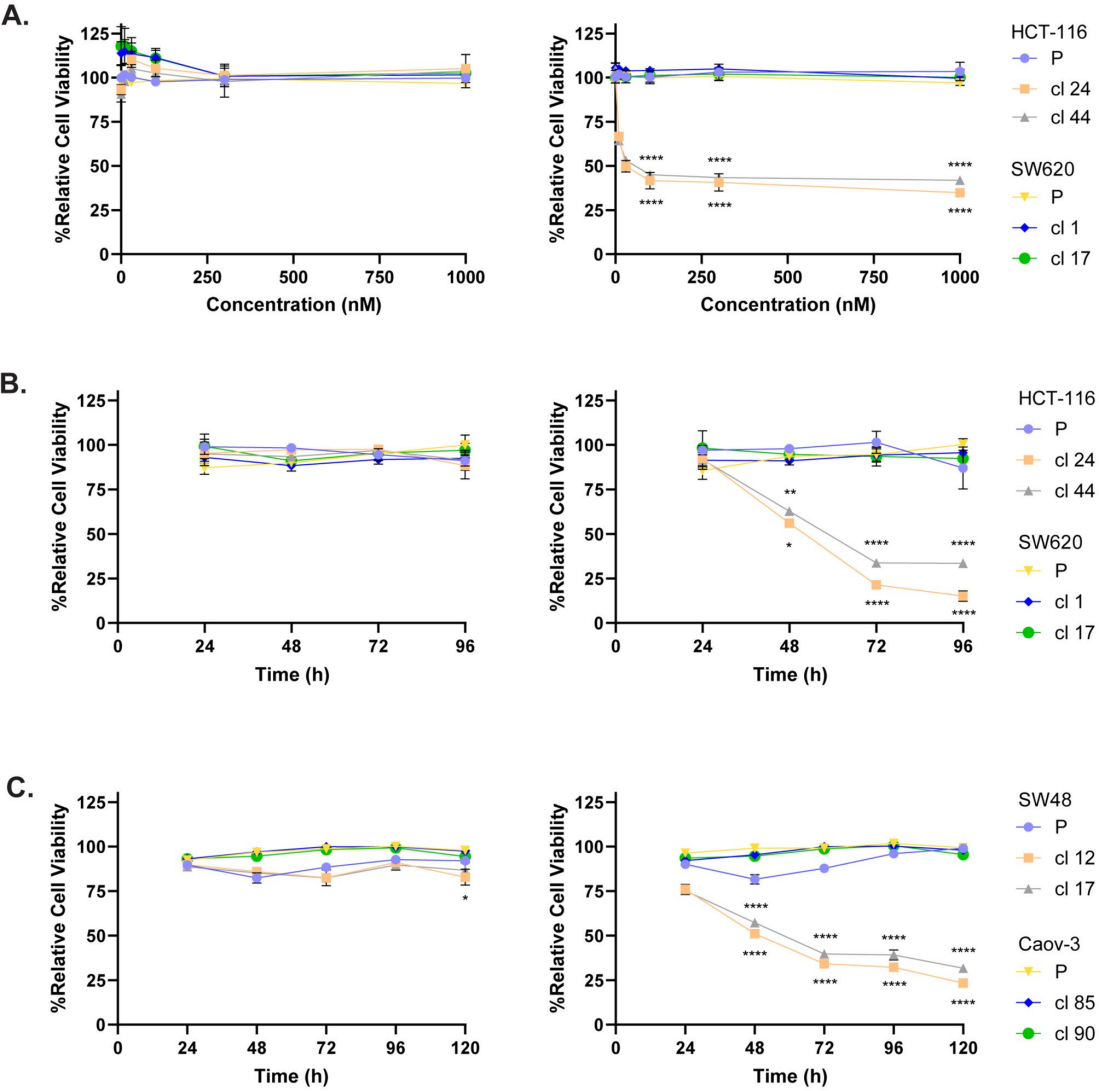
WRN degradation is selectively toxic in MSI cancer cells. Cell viability was determined through MTT analysis, and “Cell Viability” was calculated by normalising values for Cis-AGB-1- or AGB-1-treated cells to DMSO treated cells. (**A**) HCT-116 parental (P) cells, and clones 24 and 44 and SW620 parental cells (P) and clones 1 and 17 were treated with increasing concentrations of Cis-AGB-1 (left) or AGB-1 (right) for 72 h and subjected to MTT assay. All values are mean ± SEM from 4 biological repeats (*n* = 4). (**B**) Same cells as in (**A**) were treated with Cis-AGB-1 (0.3 μM; left) or AGB-1 (0.3 μM; right) for 24 h, 48 h, 72 h and 96 h in a blind manner. All values are mean ± SEM from 3 biological repeats (*n* = 3). (**C**) SW48 parental (P) cells and clones 12 and 17 and Caov-3 parental (P) cells and clones 85 and 90 were treated with 0.3 μM of Cis-AGB-1 (left) or 0.3 μM AGB-1 (right) as in (**B**) except with an extra timepoint at 120 h. Datapoints shown represent mean ± SEM from 4 biological repeats (*n* = 4). Statistical significance was analysed through a one-way ANOVA with a Šidák post-test. *P < 0.05, **P < 0.01, ***P < 0.001, ****P < 0.0001.

We next investigated the impact of conditional WRN degradation on the viability of MSI and MSS cell lines described above. We first compared HCT-116 (MSI) with SW620 (MSS) cells, testing parental cells and two independent clones for each cell line. When cells were subjected to increasing concentrations of AGB-1, but not Cis-AGB-1, for 72 h, HCT-116 Bd-WRN clones 24 and 44 showed a statistically significant, dose-dependent decrease in cell viability, with a maximal cytotoxic effect observed at around 100 nM (Fig. 3A). In contrast, AGB-1 had no significant effect on the viability of SW620 Bd-WRN clones analysed in parallel, or on parental HCT-116 cells (Fig. 3A). Blinded time course experiments (see “Materials and methods”) revealed that the viability of HCT-116 Bd-WRN clones had decreased significantly 48 h after AGB-1 exposure with maximal effects at 72 h, whereas SW620 cells were unaffected (Fig. 3B). Similar results were obtained when the Bd-WRN MSI cell line SW48 was compared with the Bd-WRN MSS cell line Caov-3 (Fig. 3C). Taken together, these data show that conditional PROTAC-mediated WRN degradation is a valid approach for treating MSI tumours.

### Conditional WRN degradation results in DNA double strand breaks, cell cycle arrest and DNA damage signalling in MSI cells but not MSS cells

**Fig. 4:**
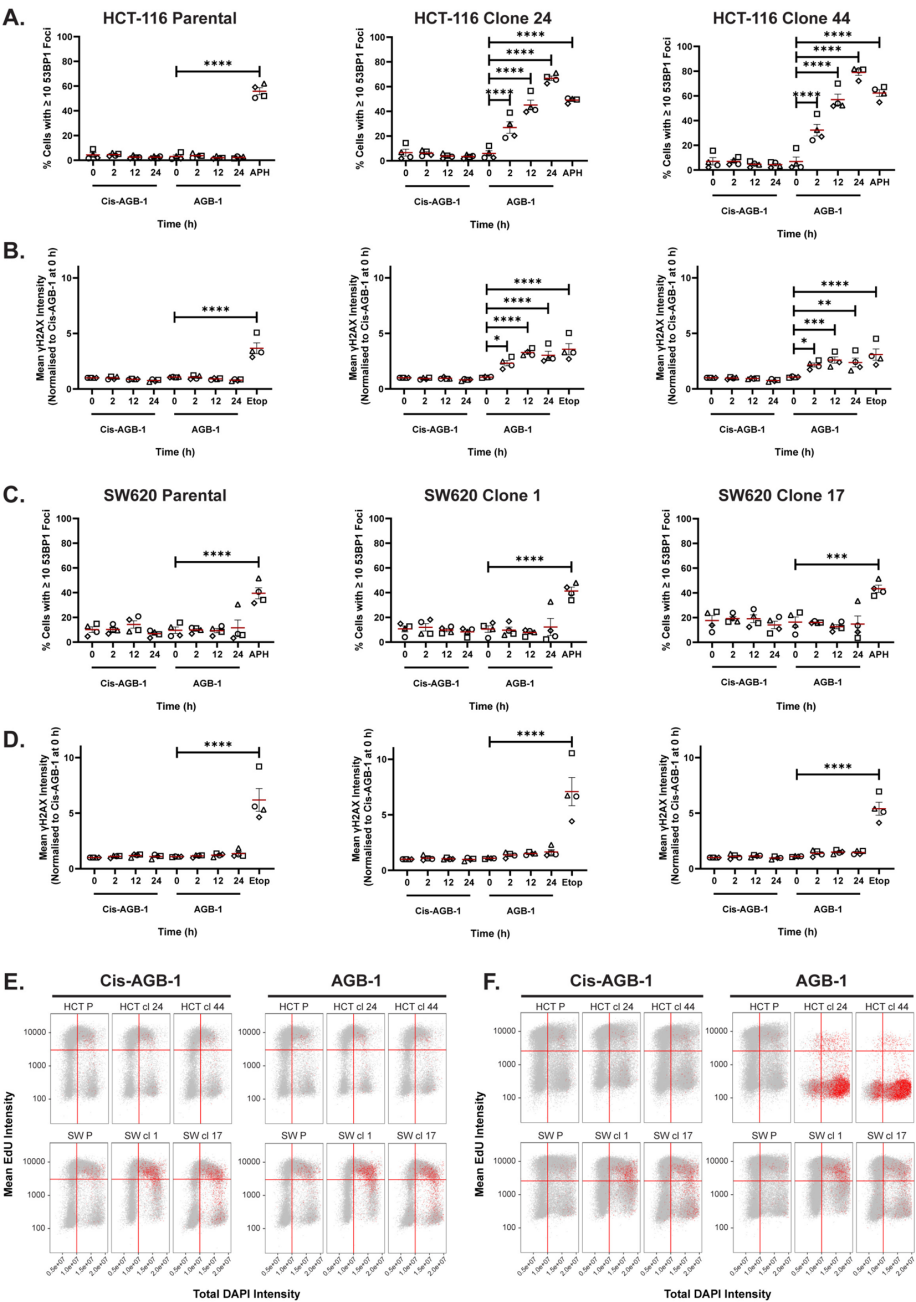
WRN degradation in MSI, but not MSS, cells causes chromosome breaks. (**A**) Quantification of the % cells with ≥ 10 53BP1 foci in HCT-116 parental cells, clones 24 and 44. Cells were treated for 0 h, 2 h, 12 h or 24 h with Cis-AGB-1 (0.3 μM) or AGB-1 (0.3 μM) and pulsed with EdU for 30 min before the end of each timepoint. Aphidicolin (0.5 μM) treatment for 24 h was used as a positive control for 53BP1 foci number. (**B**) Quantification of mean γH2AX intensity in the same cells as in (**A**). Here, cells were treated with etoposide (25 μM) for 2 h as a positive control for γH2AX foci formation. Mean intensities for all timepoints and conditions were normalised to the value for Cis-AGB-1 at 0 h. Data are represented as mean ± SEM from four biological repeats (*n* = 4) with each repeat represented by a different symbol. (**C**), (**D**) Quantification of % cells with ≥ 10 53BP1 foci (**C**) and mean γH2AX intensity (**D**), in SW620 parental cells, clones 1 and 17. Statistical significance was analysed by comparing all timepoints to the 0 h timepoint within each treatment group (Cis-AGB-1 or AGB-1) through a one-way ANOVA with a Šidák post-test. *P < 0.05, **P < 0.01, ***P < 0.001, ****P < 0.0001. (**E**) Representative cell cycle analysis, from one biological repeat of HCT-116 parental cells, clones 24 and 44 and SW620 parental cells, clones 1 and 17 treated with Cis-AGB-1 (0.3 μM) or AGB-1 (0.3 μM) at 0 h. (**F**) Same as in (**E**) after 24 h of treatment. Each dot represents a single cell; colour-coding (red) indicates cells with more than 10 53BP1 foci. P indicated parental cells.

To investigate the basis for cell death upon conditional WRN degradation in MSI cells, we checked genome integrity by measuring the levels of DNA double-strand breaks (DSBs). We used the formation of 53BP1 and γH2AX subnuclear foci as surrogate markers of DSBs^49–51^. We observed a striking, time-dependent increase in the proportion of cells with more than ten 53BP1 foci in both HCT-116 Bd-WRN clones (24 and 44) treated with AGB-1 but not Cis-AGB-1, reaching a maximum between 12 and 24 h (Fig. 4A). The maximal effect observed was similar in intensity to the effect of treating cells with low-dose aphidicolin that causes DNA replication stress and increased 53BP1 foci^52^. AGB-1 did not affect 53BP1 foci number in parental HCT-116 cells (Fig. 4A) or in SW620 cells expressing Bd-WRN (Fig. 4C). Similar results were obtained when γH2AX was used as a surrogate marker of DSBs (Fig. 4B, D). Representative images, from one biological repeat, used for γH2AX intensity and 53BP1 foci quantification after AGB-1 treatment in HCT-116 and SW620 Bd-WRN cells are shown in Figs. S5A and B. Labelling of cells with EdU revealed that after 24 h of AGB-1 treatment, most HCT-116 Bd-WRN cells are no longer in S-phase, instead accumulating in G_2_/M phases (Figs. 4E, F and S5C). Furthermore, there is a strong correlation between G_2_/M-arrested HCT-116 Bd-WRN cells and 53BP1 foci, 24 h after AGB-1 exposure (Fig. 4E, F). Increased DNA damage has been previously reported to contribute to increased nuclear size, perhaps due to resulting chromatin decondensation^28,53,54^. In accordance with this, we noted that after AGB-1 treatment, the G_2_/M-arrested population of HCT-116 Bd-WRN cells have enlarged nuclei—approximately 1.4 to 1.6 times larger nuclei than their Cis-AGB-1 treated counterparts (Fig. S5D). This was not observed in SW620 Bd-WRN cells.

**Fig. 5:**
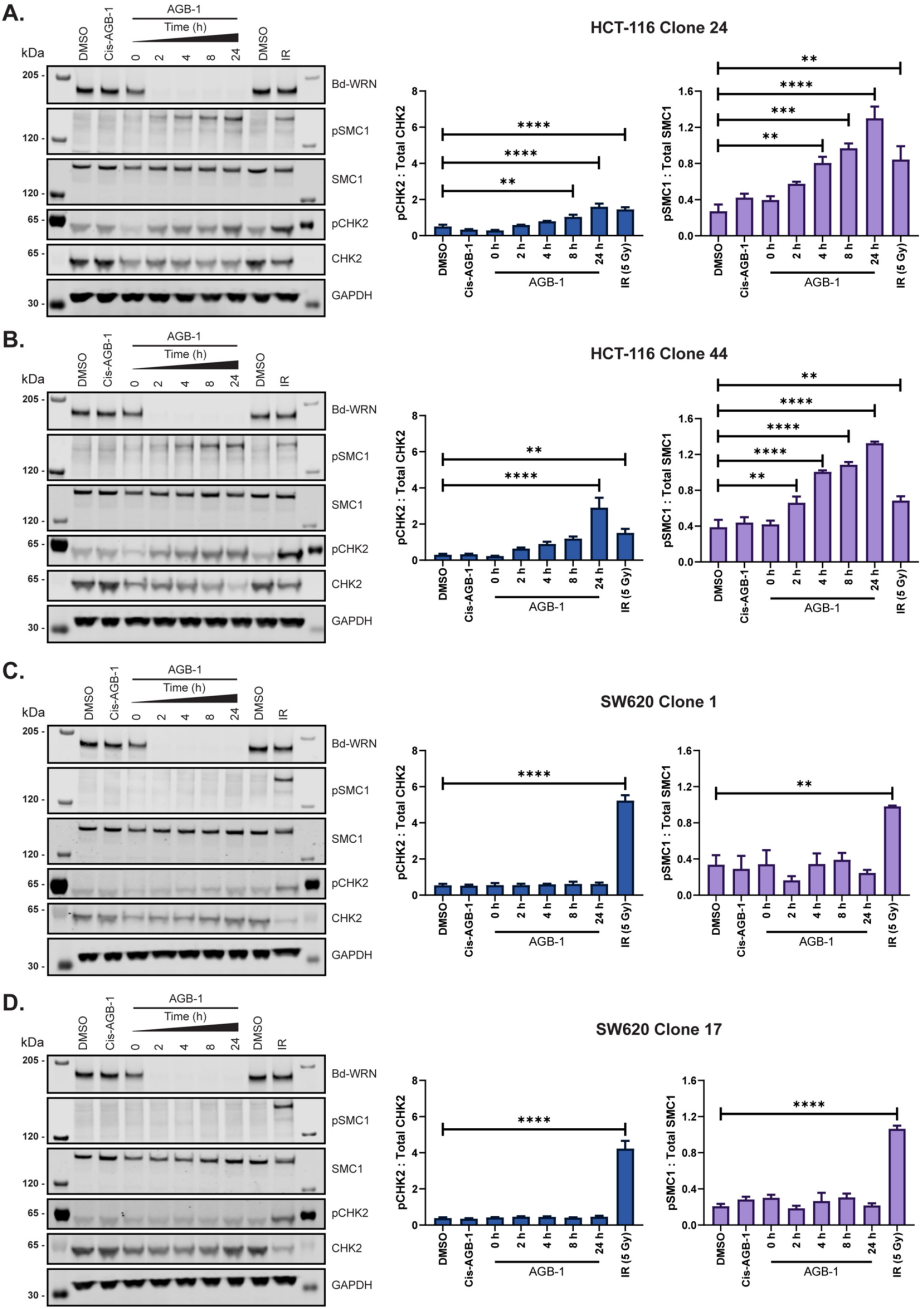
WRN degradation in MSI but not MSS, cells causes checkpoint activation. (**A**) Representative SDS-PAGE and western blot analysis of lysates from HCT-116 clone 24 treated with 0.3 μM AGB-1 for 0 h, 2 h, 4 h, 8 h or 24 h and blotted with WRN, pSMC1, total SMC1, pCHK2, total CHK2 and GAPDH, as a loading control. Blots from one of three biological repeats are shown. Cis-AGB-1 (0.3 μM) and DMSO (0.1%) treatments for 24 h were used as negative controls for WRN degradation. Cells treated with 5 grays (Gy) of ionising radiation (IR) were used as a positive control for SMC1 and CHK2 phosphorylation. Quantification (mean ± SEM) from all three repeats (*n* = 3) are shown alongside with pCHK2: total CHK2 (left) and pSMC1: total SMC1 (right). (**B**), (**C**), (**D**) same as in (**A**) for HCT-116 clone 44, SW620 clone 1 and SW620 clone 17, respectively. Statistical significance was analysed through a one-way ANOVA with a Dunnett post-test. *P < 0.05, **P < 0.01, ***P < 0.001, ****P < 0.0001.

The ATM-CHK2 DNA damage response pathway is activated in response to DSBs^51,55^. Additionally, ATM mediated phosphorylation of SMC1 at Ser^957^ and Ser^966^ is a key event that drives pro-survival responses in cells that have accumulated DSBs^56^. Two HCT-116 Bd-WRN clones examined showed a significant time dependent increase in the levels of pCHK2 pThr^68^ and SMC1 pSer^966^, known markers of ATM activation, after exposure to AGB-1 but not Cis-AGB-1, with maximal induction evident at around 24 h (Figs. 5A, B and S6A, B). AGB-1 treatment had no significant effect on the levels of pCHK2 and pSMC1 in SW620 cells (Figs. 5C, D, and S6C, D). Taken together, the data above show that conditional WRN degradation caused DNA breakage, G_2_/M arrest, increased nuclear size, and DNA damage signalling in MSI but not MSS cells.

### A cell-based assay for monitoring the selective killing of MSI versus MSS cells

**Fig. 6:**
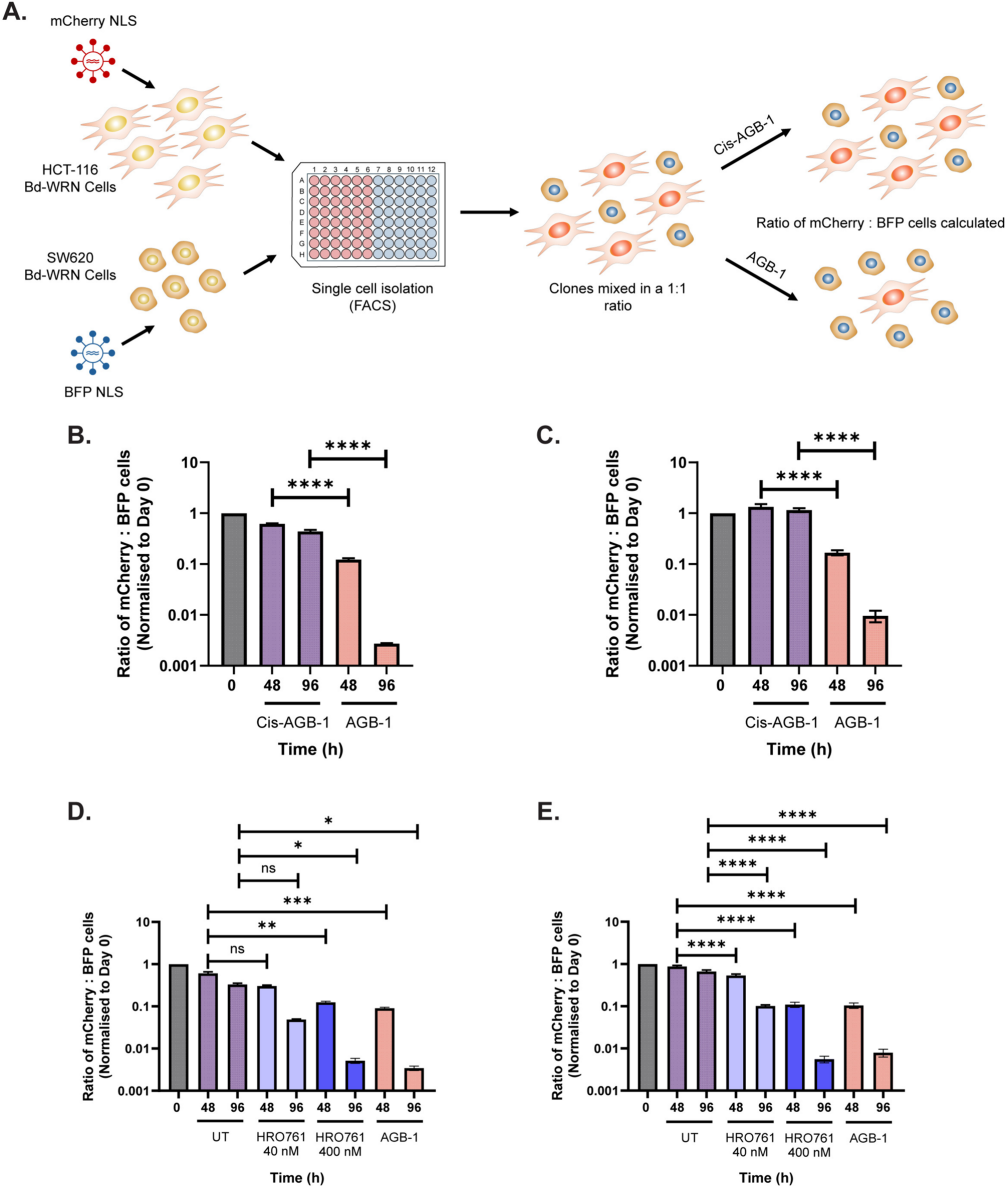
A multicolour cell-based platform for screening compound toxicity in MSI versus MSS cells. (**A**) Schematic representation of the multicolour competition assay (MCA). HCT-116 and SW620 Bd-WRN clones were transduced with lentiviruses carrying either a mCherry or BFP nuclear localisation signal (NLS), respectively. Single cell clones were isolated from transduced pools via FACS. mCherry or BFP Bd-WRN clones were then mixed in a 1:1 ratio and the ratio of mCherry to BFP cells was measured at 0 h, 48 h and 96 h after treatment with either 0.3 μM Cis-AGB-1 or AGB-1. (**B**) Graph representing the ratio of mCherry HCT-116 Bd-WRN clone 24 to BFP SW620 Bd-WRN clone 1 at 0 h, 48 h and 96 h after treatment with 0.3 μM Cis-AGB-1 or 0.3 μM AGB-1 from four biological repeats (*n* = 4). Ratios at each timepoint were normalised to ratios at 0 h. (**C**) Graph representing the same as in (**B**) with mCherry HCT-116 Bd-WRN clone 44 and BFP SW620 Bd-WRN clone 17. (**D**) Graph representing the ratio of mCherry HCT-116 Bd-WRN clone 24 to BFP SW620 Bd-WRN clone 1 at 0 h, 48 h and 96 h after treatment with 0.1% DMSO (UT), 0.3 μM AGB-1 or 40 nM and 400 nM HRO761 from four biological repeats (*n* = 4). Ratios at each timepoint were normalised to ratios at 0 h. (**E**) Graph representing the same as in (**D**) with mCherry HCT-116 Bd-WRN clone 44 and BFP SW620 Bd-WRN clone 17. Data shown are means ± SEM and statistical significance was analysed through a one-way ANOVA with a Šidák post-test. *P < 0.05, **P < 0.01, ***P < 0.001, ****P < 0.0001.

We sought to develop a method that would allow medium to high throughput screening of early stage WRN PROTACs—and other small molecule inhibitors—for selective toxicity in MSI versus MSS cells. To this end, we exploited the multi-colour cell-based assay (MCA) developed by others^57^, and adapted it to our purposes. To achieve this, HCT-116 (MSI) and SW620 (MSS) Bd-WRN cells were transduced with lentiviral vectors to induce stable expression of nuclear mCherry and BFP, respectively (Fig. 6A). mCherry HCT-116 MSI clones and BFP SW620 MSS clones were then isolated from the transduced pools via FACS and sensitivity of these clones to AGB-1 was verified by western blot (Fig. S7A–D). Clones were then mixed in a 1:1 ratio and grown for 48 h and 96 h in the presence of either Cis-AGB-1 or AGB-1, at which point, the ratio of mCherry to BFP cells was measured by flow cytometry. In the first experiment, mCherry HCT-116 Bd-WRN clone 24 was mixed with BFP SW620 Bd-WRN clone 1. As shown in Figs. 6B and S8A, exposure of this mixed population to AGB-1 led to selective, time-dependent elimination of the mCherry HCT-116 Bd-WRN clone 24, evident at 48 h and 96 h. Cis-AGB1 had no such effect. Similar data were obtained when using mCherry HCT-116 Bd-WRN clone 44 and BFP SW620 Bd-WRN clone 17 (Figs. 6C and S8B). Furthermore, AGB-1 treatment resulted in an equivalent or marginally greater elimination of the mCherry HCT-116 Bd-WRN cells when compared with HRO761 (Fig. 6D, E), an allosteric WRN inhibitor with a growth inhibitory 50% (GI_50_) of 40 nM, currently in phase I clinical trials for MSI colorectal cancers^58^. Therefore, the multicolour platform can be used for screening compounds, or gene deletions through CRISPR screens, that are selectively toxic in MSI cells compared with MSS cells.

## Discussion

WRN helicase inhibitors are currently in clinical trials for treatment of solid tumours with MSI. In this study, we demonstrated that conditional degradation of WRN is a promising alternative approach for targeting WRN in MSI cancers. PROTAC-mediated WRN degradation had a selective and potently toxic effect on MSI cells compared with MSS cells, leading to high levels of chromosome breakage, G_2_/M phase arrest, activation of DNA damage signalling and increased nuclear size. These results make a strong case for screening compound libraries for ligands that bind to WRN to enable the development of WRN-specific PROTACs for the treatment of MSI cancers. Our data are consistent with another report that was published while the current study was in progress, which employed a D-tag to achieve conditional WRN degradation^59^.

While small molecule inhibitors dampen enzymatic activity through stoichiometric targeting of enzyme active sites, PROTACs act in a catalytic manner because they can participate in multiple successive rounds of target degradation^41^. Besides this feature, there are a range of other advantages to PROTACs such as enhanced target selectivity, and a decreased likelihood of mutation-induced drug resistance. Recent studies have shown that PROTACs specific for the oestrogen receptor^60^ or for BET domain proteins^61–63^ markedly attenuate cancer progression in xenograft models. Furthermore, PROTACs that target receptor tyrosine kinases result in stronger inhibition of cancer cell proliferation compared to small molecule activity-based kinase inhibitors^64^. From this point of view, it would be highly desirable to develop a WRN-specific PROTAC. We have developed a multi-colour assay platform that will enable facile, high-throughput testing of candidate WRN PROTACs for selective toxicity towards MSI versus MSS cells. This platform could be used, in principle, to carry out genome wide CRISPR screening for alternative targets to WRN that could be used to selectively kill MSI cancer cells. Regulators of WRN function fall into this category, or factors that influence the stability of the cruciform structures that arise at TA nucleotide repeats in MSI cells, for example.

It remains to be seen how well small molecule WRN helicase inhibitors perform in clinical trials, and whether off target effects will lead to toxicity. Either way, drug resistance could be a major issue for the clinical use of WRN helicase inhibitors; the major limitation of PARP inhibitors, for example, is the emergence of drug resistance in tumours exposed to these drugs^65^. Not only might WRN degraders be more potent in killing MSI cancers, but they could also be used to treat MSI tumours that become resistant to helicase inhibitors.

## Materials and methods

### Plasmids, oligonucleotides, and antibodies

All plasmids used in this study are listed in Table 1. These plasmids were designed and synthesised by the MRC PPU, University of Dundee. Plasmid sequences, maps and constructs can be obtained from https://mrcppureagents.dundee.ac.uk/, except for the pLenti-mCherry-NLS which was obtained from^66^. Primers used for junction PCRs and genotyping clones are listed in Table 2. All primary and secondary antibodies used in this study are listed in Tables 3, 4, 5, 6.

**Table 1:** List of plasmids and CRISPR guides used in this project.

Construct	Vector
Bd-Tag WRN Anti-sense + Cas9 (D10A)	pX335
Bd-Tag WRN Donor	pMK
Bd-Tag WRN Sense + Puro	pBABED
BFP-NLS	pLenti
mCherry-NLS^66^	pLenti
VSV-G	pCMV
VSV-GP (lenti gag and pol)	pCMV

**Table 2:** List of primers used in this project.

Primer	Direction	Sequence (5′–3′)
Bd-Tag	Fwd	GCATCCTCAAGGAGATGTTTGCC
	Rev	ACTTGATTGTGCTCATGTCCATGG
EGFP	Fwd	GCATCAAGGTGAACTTCAAGATCCG
	Rev	CTCGTTGGGGTCTTTGCTCAGG
IRES	Fwd	CTGTCTTCTTGACGAGCATTCCTAGG
	Rev	CTTGCATTCCTTTGGCGAGAGG
M13	Fwd	GTAAAACGACGGCCAGTG
	Rev	GGAAACAGCTATGACCATG
WRN Nter	Fwd	ACTTGAATTTTGGTTTACATTGAGGAGTC
	Rev	GATCCAGTGAATTCTAAGAAGGGGAGG
WRN Nter2	Fwd	AGTATGAGTCATATCAGGGTACGGATCC
	Rev	TCAAAAACACTCTTCCGAACACATGC

**Table 3:** List of primary antibodies used for western blots in this project.

Target Protein/Name	Species	Dilution	Manufacturer	Catalogue No
CHK2 (D9C6)	Rabbit	1:5000	Cell Signalling	6334S
GAPDH	Rabbit	1:10,000	Cell Signalling	2118S
Phospho CHK2 (Thr68)	Rabbit	1:5000	Cell Signalling	2661S
Phospho SMC1 (S966)	Rabbit	1:5000	Bethyl	A300-050A
SMC1 (8E6)	Mouse	1:5000	Cell Signalling	6892S
WRN Monoclonal [195C]	Mouse	1:5,000	Abcam	Ab241545
α-Tubulin (DM1A)	Mouse	1:10,000	Cell Signalling	3873S

**Table 4:** List of primary antibodies used for immunofluorescence in this project.

Target Protein/Name	Species	Dilution	Manufacturer	Catalogue No
53BP1	Rabbit	1:5000	Novus Bio	NB100-304
Phospho γH2AX (S139)	Mouse	1:2000	Merck	05–636

**Table 5:** List of secondary antibodies used for western blots in this project.

Name	Species	Dilution	Manufacturer	Catalogue No.
Anti-Mouse 680	Goat	1:10,000	LI-COR	926-68070
Anti-Mouse 680	Donkey	1:10,000	LI-COR	926-68072
Anti-Mouse 800	Goat	1:10,000	LI-COR	926-32210
Anti-Mouse 800	Donkey	1:10,000	LI-COR	926-32212
Anti-Rabbit 680	Goat	1:10,000	LI-COR	926-68071
Anti-Rabbit 680	Donkey	1:10,000	LI-COR	926-68073
Anti-Rabbit 800	Goat	1:10,000	LI-COR	926-32211
Anti-Rabbit 800	Donkey	1:10,000	LI-COR	926-32213

**Table 6:** List of secondary antibodies used for immunofluorescence in this project.

Name	Species	Dilution	Manufacturer	Catalogue No.
Anti-Mouse Alexa Fluor™ 488	Donkey	1:1000	Thermo Fisher	A-21202
Anti-Rabbit Alexa Fluor™ 546	Goat	1:1000	Thermo Fisher	A-11035

### Cell culture

HEK293 FT cells, and HCT-116, SW620 and SW48 colorectal cancer cells were obtained from the MRC PPU stocks at University of Dundee. Caov-3 ovarian cancer cells were kindly provided by Gillian Smith, Jacqui Wood Cancer Centre, Dundee. HEK293 FT, SW620 and SW48 cells were cultured in DMEM (Sigma). HCT-116 and Caov-3 cells were maintained in McCoy’s 5A (sigma) and RPMI (Gibco) media, respectively. All media were supplemented with 10% (v/v) foetal bovine serum (FBS) (Gibco), 1% (v/v) penicillin/streptomycin (pen/strep) (Gibco) and 2 mM L-glutamine (Gibco). DMEM was also supplemented with 1 mM sodium pyruvate (Gibco) and 1X Non-Essential Amino Acids (NEAA) (Gibco). RPMI was supplemented with 20% (v/v) FBS. All cells were grown at 37 °C, 5% CO_2_, and 95% humidity. Cells were regularly checked to be negative for Mycoplasma contamination.

### Generating Bd-tagged WRN knock-in cell lines

Approximately 1 × 10^6^ HCT-116, SW620, SW48 and Caov-3 cells were seeded in 10 cm cell culture dishes 24 h prior to transfection. Cells were transfected using 20 μg PEI (Polysciences), 3 μg of the pMK vector containing the knock-in EGFP-IRES-BdTag-WRN donor sequence, 1 μg of the px335 vector containing the antisense sgRNA (5′-TTCTGCACATTCATCCATTC) and spCas9n (D10A) expression cassette and 1 μg of the pBabeD P U6 Puro vector containing the sense sgRNA (5′-ATGTGCTGTAGAAGAAAGAA) and puromycin resistance expression cassette (Table 1). The pMK donor plasmid consisted of a pair of homology arms 518 bp upstream and 594 bp downstream of the N-terminal WRN insertion site. The next day, 2 μg/ml of puromycin was added to cell media to begin selection. After 48 h of puromycin selection, cells were washed with PBS and replenished with fresh media without puromycin. Cells were allowed to recover in normal media for 1 week prior to cell sorting.

### Single cell cloning

Genome edited knock-in cell pools were harvested using 0.05% Trypsin–EDTA (Gibco) and resuspended in DMEM supplemented with 1% FBS and 100 μg/ml Normocin (Invivogen) to a concentration of approximately 5 × 10^6^ cells/ml. Fluorescence Activated Cell Sorting (FACS) was performed using an MA900 cell sorter (Sony Biotechnology), equipped with a 130nm nozzle. Forward angle light scatter (FSC) and back scatter (BSC) were generated using a 488nm laser and detected using 488 ± 17nm band pass filters. Cells were distinguished from debris based on FSC-Area(A) and SSC-A measurements. Single cells were distinguished from doublets and clumps based on FSC-A and FSC-Width (W) measurements. Co-linear 488nm laser was used to excite GFP and fluorescence was detected using a 525 ± 50nm band pass filter. 561nm and 405nm lasers were used to excite mCherry and BFP and fluorescence was detected using 617 ± 30 and 450 ± 50 band pass filters, respectively. Positive cells were identified by assessing the background autofluorescence of control (untransfected) cells which did not express fluorescent proteins. Single positive cells were sorted into individual wells of a 96-well plate containing 200 μl of 50% pre-conditioned media obtained from healthy cells and 50% fresh media (20% FBS) according to the cell type. Due to low efficiency of transfection with the Bd-WRN SW48 cells, transfected cell pools were bulk sorted for GFP expression first using the standard semi-purity mode, expanded, and re-sorted for GFP to isolate single cell clones as above. After FACS, plates were spun at 1000 rpm for 3 min and clones were left to grow at 37 °C, 5% CO_2_, and 95% humidity for 3 weeks. All surviving colonies were expanded and analysed via western blotting and PCR strategies to detect Bd-WRN or through flow cytometry to detect mCherry and BFP clones.

### Cell lysis and protein quantification

Cells were lysed in ice cold RIPA buffer comprised of 50 mM Tris–HCl (pH 8.0), 1 mM EDTA, 0.5 mM EGTA, 0.1% SDS, 0.1% Sodium deoxycholate, 150 mM NaCl, 0.5 U/ml Pierce Universal Nuclease (Thermo Fisher), 1:10,000 Microcystin-Lr (Enzo), 1:100 Phosphatase Inhibitor Cocktail (Sigma-Aldrich) and 1 × Complete EDTA-free Protease Inhibitor (Roche). Lysates were incubated on ice for 30 min and then cleared by centrifugation at 17,000 xg for 10 min. Protein concentration was estimated using the Pierce™ BCA Protein Assay kit (Thermo Fisher) according to the manufacturer’s instructions.

### SDS-PAGE and western blotting

Lysates were incubated with 1/4 volume 4X NuPAGE® LDS Sample Buffer (Invitrogen) supplemented with 2.5% (v/v) β-mercaptoethanol and boiled at 95 °C for 5 min. Protein samples were separated by SDS-PAGE (50 μg/well) using NuPAGE® 3–8% Tris–Acetate gels (Thermo Fisher) at 110V for 1 h and 10 min. PageRuler™ Prestained Protein Ladder (Thermo Fisher) was used as a marker for molecular weight. Proteins were then transferred onto an Amersham Protran 0.45 μM nitrocellulose membrane (Cytiva) at a constant voltage of 90V for 1.5 h using 25 mM Tris, 192 mM Glycine and 20% Methanol. Membranes were then blocked for 30 min in 5% milk TBS-T (20 mM Tris, 150 mM NaCl, 0.2% Tween® 20 (v/v)). Protein detection was carried out by incubating primary antibodies (Table 3), diluted in 5% milk TBS-T for non-phospho antibodies or 5% BSA TBS-T for phospho antibodies, overnight at 4 °C. The next day, membranes were washed thrice for 5 min in TBS-T before incubating with secondary antibodies (Table 5), diluted in 5% milk TBS-T for non-phospho antibodies or 5% BSA TBS-T for phospho antibodies, for 1 h at RT. Membranes were then washed thrice for 5 min in TBS-T, imaged using Odyssey CLx Scanner (LI-COR) and subsequently analysed in Empiria Studio v. 2.3 (LI-COR). When indicated, quantification of western blot bands was performed using ImageJ (v1.53), normalizing the intensity of the relevant target protein band to the corresponding loading control.

### Genomic DNA extraction

Cells were pelleted at 1500 rpm for 5 min and genomic DNA was extracted using the DNeasy Blood & Tissue Kit (Qiagen) according to the manufacturer’s instructions. DNA concentration was estimated on NanoDrop™ Spectrophotometer (Thermo Fisher).

### Junction PCR amplification of targeted *WRN* locus

The knock-in (KI) target region for parental or Bd-WRN HCT-116, SW620, SW48 and Caov-3 clones was amplified using 300 ng of DNA template and the PrimeSTAR® GXL DNA Polymerase (Takara Bio) according to the manufacturer’s instructions using a C1000 Touch Thermal Cycler (BioRad). Primers used included the Nter2 flanking primers (binding outside of the homology regions) or Nter2 Fwd and GFP Rev or bdTAG Fwd and Nter2 Rev (Table 2).

### Agarose gel electrophoresis

10 μl of PCR products were mixed with 1/6 volume Gel Loading Dye, Purple (6x) (NEB) and loaded on a 1% (w/v) agarose gel in Tris–acetate-EDTA (TAE) buffer (40 mM Tris, 20 mM acetic acid, 1 mM EDTA, pH 8.0) with 1X SYBR™ Safe DNA Gel Stain (Thermo Fisher). 7 μl of 1 kb Plus DNA Ladder (Thermo Fisher) was used as a marker for size of DNA samples. DNA samples were separated through electrophoresis in TAE buffer at 100V for 45 min and gels were imaged using U:Genius^2^ Gel documentation system (Syngene).

### Genotyping Bd-WRN knock-in clones

Following junction PCR amplification of the KI target region, PCR products generated with the Nter2 flanking primers (Table 2) were ligated into the pSC-B-amp/kan blunt end cloning vector using the StrataClone Blunt PCR Cloning Kit (Agilent), according to manufacturer’s instructions. Ligated vectors were transformed into the provided Cre recombinase proficient StrataClone SoloPack competent cells, plated on 100 μg/ml ampicillin agar plates and incubated at 37 °C overnight. The next day, 12–15 single colonies were picked per KI cell clone and grown overnight in 5 ml LB media supplemented with 100 μg/ml ampicillin at 37 °C, shaking. After 24 h, bacterial cultures were pelleted at 5000 rpm for 15 min and sent to DNA Sequencing and Services (University of Dundee) for genomic DNA minipreps. Subsequently, DNA sequencing was performed by DNA Sequencing & Services (MRC PPU, School of Life Sciences, University of Dundee, Scotland, www.dnaseq.co.uk) using Applied Biosystems Big-Dye Ver 3.1 chemistry on an Applied Biosystems model 3730 automated capillary DNA sequencer using M13 F and R, Bd-Tag R and IRES R primers (Table 2).

### Dose–response WRN degradation assays

After genotypic and western blot analysis, 5 × 10^5^ parental or Bd-WRN clones were plated in 6-well plates in 2 ml of cell culture media and grown at 37 °C, 5% CO_2_, and 95% humidity. 24 h later, AGB-1 (Tocris Bioscience) reconstituted in DMSO was added to the cells at the following final concentrations: 1000 nM, 300 nM, 100 nM, 30 nM, 10 nM, 3 nM, and 1 nM. Each clone and parental cells were also incubated with 0.1% DMSO and 1000 nM Cis-AGB-1 (Tocris Bioscience) as controls. Cells were treated for 3 h and then harvested with 0.05% Trypsin–EDTA (Gibco), lysed and analysed via western blotting.

### Time course WRN degradation assays

Approximately 5 × 10^5^ parental or Bd-WRN clones were plated in 6-well plates in 2 ml of cell culture media and grown at 37 °C, 5% CO_2_, and 95% humidity. 24 h later, AGB-1 was added to cells at a final concentration of 300 nM. Each Bd-WRN clone and parental cells were also incubated with 0.1% DMSO and 300 nM Cis-AGB-1. Cells were harvested at 0 h, 0.5 h, 1 h, 2 h, 4 h and 8 h after treatment, lysed and analysed via western blotting. DMSO and Cis-AGB-1 treated cells were harvested after 8 h of treatment only.

### Proteasome dependence of Bd-WRN degradation

Approximately 5 × 10^5^ parental or Bd-WRN clones were plated in 6-well plates in 2 ml of cell culture media and grown at 37 °C, 5% CO_2_, and 95% humidity. Clones analysed either had untagged and Bd-tagged WRN or only Bd-tagged WRN. 24 h later, cells were pre-treated with either 3 μM of MLN4924 (Active Biochem), 50 μM of MG132 (Sigma) or 0.1% DMSO for 1 h. Pre-treated cells were then incubated with either 300 nM AGB-1, 300 nM Cis-AGB-1 or 0.1% DMSO in the presence of MLN4924 or MG132 for a further 3 h. Cells were then harvested with 0.05% Trypsin–EDTA (Gibco), lysed and analysed via western blotting.

### Cell viability assays

Viability assays were conducted in pairs comprised of one MSI and one MSS cell line—HCT-116 and SW620 cells were the first pair and SW48 and Caov-3 were the second pair. For each MSI-MSS pair, between 2 × 10^3^ and 5 × 10^3^ parental and two Bd-WRN clones per cell type were plated in duplicate, in a 96-well plate in 50 μl of their respective cell culture media and grown at 37 °C, 5% CO_2_, and 95% humidity. For dose response assays, 24 h after plating, 50 μl of media supplemented with 2X DMSO (0.1% final) or 2X concentrations of AGB-1 and Cis-AGB-1 at final concentrations ranging from 1000 nM, 300 nM, 100 nM, 30 nM, 10 nM, and 0 nM were added to the relevant wells. Cells were then incubated at 37 °C, 5% CO_2_, and 95% humidity for 72 h, after which, cell viability was assessed. For time course assays, 24 h after plating, 50 μl of media supplemented with 2X DMSO (0.1% final) or 2X AGB-1 (300 nM final) and Cis-AGB-1 (300 nM final) were added to relevant wells. Cells were then incubated at 37 °C, 5% CO_2_, and 95% humidity for 24 h, 48 h, 72 h and 96 h. The SW48 and Caov-3 pair was grown till an extra timepoint of 120 h. DMSO, AGB-1 Cis-AGB-1 was replaced in plates every 48 h. Time course cell viability assays were performed in a blind manner, with the identities of DMSO, Cis-AGB-1 and AGB-1 anonymised as compounds 1, 2 and 3 by other lab members. To measure cell viability at each time point, 20 μl/well of CellTiter®-Glo 2.0 Cell Viability Assay Reagent (Promega) was added per well. Plates were gently shaken and incubated at 37 °C, 5% CO_2_, and 95% humidity for 2.5 h. Absorbance at 490 nm (A_490_) was then measured on the Epoch microplate spectrophotometer (BioTek). Raw absorbance values were converted to percentage cell viability by normalising AGB-1 and Cis-AGB-1 readouts to the DMSO control condition for each respective cell type at each concentration or timepoint.

### Investigating checkpoint activation (pCHK2 and pSMC1 analysis)

5 × 10^5^ Bd-WRN HCT-116 and SW620 clones were plated in 6-well plates in 2 ml of cell culture media and grown at 37 °C, 5% CO_2_, and 95% humidity. 24 h later, AGB-1 was added to cells at a final concentration of 300 nM. Each clone was also incubated with 0.1% DMSO and 300 nM Cis-AGB-1 as controls. Cells were harvested at 0 h, 2 h, 4 h, 8 h and 24 h after treatment, lysed and analysed via western blotting. DMSO and Cis-AGB-1 treated cells were harvested after 24 h only. Immediately after adding compounds, a separate plate of cells were irradiated with a 36.52 TBq Cesium-137 ionising radiation (IR) source with a dose of 5 grays (Gy) as a positive control for checkpoint activation. After irradiation, cells were returned to the incubator for 30 min before harvesting. All cells were then lysed and analysed via western blotting.

### Immunofluorescence

2 × 10^4^ HCT-116 parental and Bd-WRN clones and 3 × 10^4^ SW620 parental and Bd-WRN clones were seeded in triplicate in CELLSTAR μClear 96-well plates (Greiner) with 50 μl of media. The next day, 50 μl of media supplemented with 2X of compounds AGB-1 (300 nM final), Cis-AGB-1 (300 nM final), Etoposide (25 μM final) (Sigma) or Aphidicolin (0.5 μM final) (Thermo Fisher) were added to the relevant wells. Cells were treated for 0 h, 2 h, 12 h or 24 h; Etoposide and Aphidicolin treatments were done for 2 h and 24 h, respectively. During the last 30 min of each timepoint, cells were incubated with 10 μM EdU. Cells were then fixed with 100 μl/well of 4% paraformaldehyde (Thermo Fisher) for 15 min at RT and then permeabilised with 100 μl/well of 1% Triton X-100-PBS for 5 min. Click-IT reaction was performed for 90 min with 100 μl/well of 10 mM CuSO_4_, 1.875 μM AlexaFluor™ 647-Azide (Thermo Fisher) and 10 mM Ascorbic acid made up in PBS. Cells were washed and blocked for 1 h with 100 μl/well blocking buffer (PBS, 1% BSA, 0.2% Triton X-100). Primary antibodies for γH2AX and 53BP1 (Table 4) were made up in blocking buffer, and 70 μl/well was added to wells and left overnight at 4 °C. The next day, wells were washed, and relevant secondaries (Table 6) were made up with 10 μg/ml of DAPI in blocking buffer and 70 μl/well added to wells for 1 h. Wells were washed, and then imaged using an Olympus ScanR high-content automated microscope with a × 20 objective. For each well, 25 fields were imaged and between 2000–12,000 cells per condition. Analysis was performed using the Scan-R analysis software and graphs were generated using GraphPad Prism 10 or with R Statistical Software (V 4.1.0, R Core team, 2021) using the ggplot library for visualisation.

### Lentiviral production and transduction

2 × 10^6^ HEK293 FT packaging cells were plated in a 10 cm dish and the next day, cells were transfected with 30 μg PEI, 3.25 μg VSV-GP (MRC PPU, University of Dundee), 1.75 μg VSV-G (MRC PPU, University of Dundee) and 5 μg of either pLenti-mCherry-NLS or pLenti-BFP-NLS (Table 1). pLenti-mCherry-NLS was obtained from^66^. *Age*I and *Bam*HI were used to replace mCherry-NLS with a TagBFP-NLS construct to generate the pLenti-BFP-NLS plasmid. After 24 h, 5 × 10^5^ Bd-WRN HCT-116 and SW620 clones were seeded in 6-well plates and media of the packaging cells were replaced with media of target cell line to be transduced. The next day, target cells were treated with 8 μg/ml of polybrene (Merck) for 2 h and 0, 0.1, 0.5, 1, 1.5 or 2 ml of filtered viral supernatant was added to target cells to a final volume of 2 ml, diluted with media. Transduced pools were expanded, and single cell clones were isolated through FACS based on BFP or mCherry expression.

### Multicolour assay (MCA)

Either mCherry HCT-116 Bd-WRN clone 24 and BFP SW620 Bd-WRN Clone 1 or mCherry HCT-116 Bd-WRN clone 44 and BFP SW620 Bd-WRN clone 17 were mixed in a 1:1 ratio (50,000 cells each) in 6-well plates and grown in DMEM media. Parental Bd-WRN clones were also plated in separate wells as controls to identify non-fluorescent cells. After 24 h, untreated cells were harvested for flow cytometry analysis and the rest of the plates were treated with either 300 nM AGB-1 and 300 nM Cis-AGB-1 or HRO761 (40 nM and 400 nM) (MedChem Express) and DMSO (0.1%). Live cells were analysed on the BD LSR Fortessa at 48 h and 96 h after adding compounds using the 405 nm and the 561 nm laser for BFP and mCherry cell detection, respectively. AGB-1, Cis-AGB-1 and DMSO were replaced every 48 h and HRO761 was replaced every 24 h.

### Statistical analysis

Statistical significance for experiments were evaluated with an ordinary one-way analysis of variance (ANOVA) in GraphPad Prism 10. When multiple comparisons were performed in pairs, the Šidák post-test was used and when comparisons were made to a single control condition, the Dunnett’s post-test was used. The *P* values from post-test comparisons and resulting statistical significance is indicated within figures and figure legends as; *P < 0.05, **P < 0.01, ***P < 0.001, ****P < 0.0001.

## Supplementary Information

Supplementary Information 1.

Supplementary Information 2.

## Data Availability

All data supporting the findings of this study are available from the corresponding author upon reasonable request.

## References

[1] Hakem, R. DNA-damage repair; the good, the bad, and the ugly. *EMBO J.* **27**, 589–605 (2008). 18285820 10.1038/emboj.2008.15 PMC2262034

[2] Jiricny, J. The multifaceted mismatch-repair system. *Nat. Rev. Mol. Cell Biol.* **7**, 335–346 (2006). 16612326 10.1038/nrm1907

[3] Jiricny, J. Postreplicative mismatch repair. *Cold Spring Harb. Perspect. Biol.* **5**, a012633. 10.1101/cshperspect.a012633 (2013). 23545421 10.1101/cshperspect.a012633 PMC3683899

[4] Tieng, F. Y. F., Abu, N., Lee, L.-H. & Ab Mutalib, N.-S. Microsatellite instability in colorectal cancer liquid biopsy—current updates on its potential in non-invasive detection, prognosis and as a predictive marker. *Diagnostics* **11**, 544 (2021). 33803882 10.3390/diagnostics11030544 PMC8003257

[5] Li, G.-M. Mechanisms and functions of DNA mismatch repair. *Cell Res.* **18**, 85–98 (2008). 18157157 10.1038/cr.2007.115

[6] Nilbert, M., Planck, M., Fernebro, E., Borg, A. & Johnson, A. Microsatellite instability is rare in rectal carcinomas and signifies hereditary cancer. *Eur. J. Cancer* **35**, 942–945. 10.1016/s0959-8049(99)00045-3 (1999). 10533476 10.1016/s0959-8049(99)00045-3

[7] Tiwari, A. K., Roy, H. K. & Lynch, H. T. Lynch syndrome in the 21st century: clinical perspectives. *QJM* **109**, 151–158 (2016). 26224055 10.1093/qjmed/hcv137

[8] Olave, M. C. & Graham, R. P. Mismatch repair deficiency: The what, how and why it is important. *Genes Chromosom. Cancer* **61**, 314–321 (2022). 34837268 10.1002/gcc.23015

[9] Hause, R. J., Pritchard, C. C., Shendure, J. & Salipante, S. J. Classification and characterization of microsatellite instability across 18 cancer types. *Nat. Med.* **22**, 1342–1350 (2016). 27694933 10.1038/nm.4191

[10] Laghi, L., Bianchi, P. & Malesci, A. Differences and evolution of the methods for the assessment of microsatellite instability. *Oncogene* **27**, 6313–6321 (2008). 18679418 10.1038/onc.2008.217

[11] Boland, C. R. & Goel, A. Microsatellite instability in colorectal cancer. *Gastroenterology* **138**, 2073-2087.e3 (2010). 20420947 10.1053/j.gastro.2009.12.064 PMC3037515

[12] Chiaravalli, A. M. *et al.* Immunohistochemical pattern of hMSH2/hMLH1 in familial and sporadic colorectal, gastric, endometrial and ovarian carcinomas with instability in microsatellite sequences. *Virchows Archiv* **438**, 39–48 (2001). 11213834 10.1007/s004280000325

[13] World Cancer Research Fund International. *Stomach Cancer Statistics*, https://www.wcrf.org/cancer-trends/stomach-cancer-statistics/ (2022). Accessed 22 May 2024.

[14] International Agency for Research on Cancer (WHO). *Global Cancer Observatory*, https://gco.iarc.fr/en (2024). Accessed 22 May 2024.

[15] Chan, E. M. *et al.* WRN helicase is a synthetic lethal target in microsatellite unstable cancers. *Nature* **568**, 551–556 (2019). 30971823 10.1038/s41586-019-1102-x PMC6580861

[16] Lieb, S. *et al.* Werner syndrome helicase is a selective vulnerability of microsatellite instability-high tumor cells. *Elife* **8**, e43333 (2019). 30910006 10.7554/eLife.43333 PMC6435321

[17] Le, D. T. *et al.* Mismatch repair deficiency predicts response of solid tumors to PD-1 blockade. *Science* **357**, 409–413 (2017). 28596308 10.1126/science.aan6733 PMC5576142

[18] Le, D. T. *et al.* PD-1 blockade in tumors with mismatch-repair deficiency. *N. Engl. J. Med.* **372**, 2509–2520 (2015). 26028255 10.1056/NEJMoa1500596 PMC4481136

[19] Overman, M. J. *et al.* Durable clinical benefit with nivolumab plus ipilimumab in DNA mismatch repair-deficient/microsatellite instability-high metastatic colorectal cancer. *J. Clin. Oncol.* **36**, 773–779 (2018). 29355075 10.1200/JCO.2017.76.9901

[20] Overman, M. J. *et al.* Nivolumab in patients with metastatic DNA mismatch repair-deficient or microsatellite instability-high colorectal cancer (CheckMate 142): An open-label, multicentre, phase 2 study. *Lancet Oncol.* **18**, 1182–1191 (2017). 28734759 10.1016/S1470-2045(17)30422-9 PMC6207072

[21] Gurjao, C. *et al.* Intrinsic resistance to immune checkpoint blockade in a mismatch repair–deficient colorectal cancer. *Cancer Immunol. Res.* **7**, 1230–1236 (2019). 31217164 10.1158/2326-6066.CIR-18-0683 PMC6679789

[22] Monjazeb, A. M. *et al.* A randomized trial of combined PD-L1 and CTLA-4 inhibition with targeted low-dose or hypofractionated radiation for patients with metastatic colorectal cancer. *Clin. Cancer Res.* **27**, 2470–2480 (2021). 33568343 10.1158/1078-0432.CCR-20-4632 PMC8102320

[23] Behan, F. M. *et al.* Prioritization of cancer therapeutic targets using CRISPR–Cas9 screens. *Nature* **568**, 511–516 (2019). 30971826 10.1038/s41586-019-1103-9

[24] Kategaya, L., Perumal, S. K., Hager, J. H. & Belmont, L. D. Werner syndrome helicase is required for the survival of cancer cells with microsatellite instability. *Iscience* **13**, 488–497 (2019). 30898619 10.1016/j.isci.2019.02.006 PMC6441948

[25] Mengoli, V. *et al.* WRN helicase and mismatch repair complexes independently and synergistically disrupt cruciform DNA structures. *EMBO J.* **42**, e111998 (2023). 36541070 10.15252/embj.2022111998 PMC9890227

[26] van Wietmarschen, N. *et al.* Repeat expansions confer WRN dependence in microsatellite-unstable cancers. *Nature* **586**, 292–298 (2020). 32999459 10.1038/s41586-020-2769-8 PMC8916167

[27] van Wietmarschen, N., Nathan, W. J. & Nussenzweig, A. The WRN helicase: Resolving a new target in microsatellite unstable cancers. *Curr. Opin. Genet. Dev.* **71**, 34–38 (2021). 34284257 10.1016/j.gde.2021.06.014

[28] Rodríguez Pérez, F. *et al.* WRN inhibition leads to its chromatin-associated degradation via the PIAS4-RNF4-p97/VCP axis. *Nat. Commun.* **15**, 6059 (2024). 39025847 10.1038/s41467-024-50178-3 PMC11258360

[29] Croteau, D. L., Popuri, V., Opresko, P. L. & Bohr, V. A. Human RecQ helicases in DNA repair, recombination, and replication. *Annu. Rev. Biochem.* **83**, 519 (2014). 24606147 10.1146/annurev-biochem-060713-035428 PMC4586249

[30] Parker, M. J. *et al.* Identification of 2-sulfonyl/sulfonamide pyrimidines as covalent inhibitors of WRN using a multiplexed high-throughput screening assay. *Biochemistry* **62**, 2147–2160 (2023). 37403936 10.1021/acs.biochem.2c00599 PMC10358344

[31] Heuser, A. *et al.* Challenges for the discovery of non-covalent WRN helicase inhibitors. *ChemMedChem* **19**, e202300613 (2024). 38334957 10.1002/cmdc.202300613

[32] Aggarwal, M., Sommers, J. A., Shoemaker, R. H. & Brosh, R. M. Jr. Inhibition of helicase activity by a small molecule impairs Werner syndrome helicase (WRN) function in the cellular response to DNA damage or replication stress. *Proc. Natl. Acad. Sci.* **108**, 1525–1530 (2011). 21220316 10.1073/pnas.1006423108 PMC3029756

[33] Nguyen, G. H. *et al.* A small molecule inhibitor of the BLM helicase modulates chromosome stability in human cells. *Chem. Biol.* **20**, 55–62 (2013). 23352139 10.1016/j.chembiol.2012.10.016 PMC3558928

[34] Aggarwal, M. *et al.* Werner syndrome helicase has a critical role in DNA damage responses in the absence of a functional fanconi anemia pathway. *Cancer Res.* **73**, 5497–5507 (2013). 23867477 10.1158/0008-5472.CAN-12-2975 PMC3766423

[35] Sommers, J. A. *et al.* A high-throughput screen to identify novel small molecule inhibitors of the Werner Syndrome Helicase-Nuclease (WRN). *PLoS ONE* **14**, e0210525 (2019). 30625228 10.1371/journal.pone.0210525 PMC6326523

[36] Picco, G. *et al.* Novel WRN helicase inhibitors selectively target microsatellite unstable cancer cells. *Cancer Discov.* **23**, B20 (2024). 10.1158/2159-8290.CD-24-0052 PMC7616858 38587317

[37] Kikuchi, S. *et al.* Abstract ND11: Chemoproteomic-enabled discovery of VVD-214, a synthetic lethal allosteric inhibitor of WRN helicase. *Cancer Res.* **84**, ND11 (2024). 10.1158/1538-7445.AM2024-ND11

[38] Cortes-Cros, M. *et al.* Abstract PR007: Discovery of HRO761, a novel, first-in-class clinical stage WRN inhibitor with potent and selective anti-tumor activity in cancers with microsatellite instability. *Mol. Cancer Therap.* **22**, PR007 (2023). 10.1158/1535-7163.TARG-23-PR007

[39] Sakamoto, K. M. *et al.* Protacs: chimeric molecules that target proteins to the Skp1-Cullin-F box complex for ubiquitination and degradation. *Proc. Natl. Acad. Sci. USA* **98**, 8554–8559. 10.1073/pnas.141230798 (2001). 11438690 10.1073/pnas.141230798 PMC37474

[40] Biopharma PEG. *PROTACs VS. Traditional Small Molecule Inhibitors*, https://www.biochempeg.com/article/233.html (2021). Accessed 22 May 2024.

[41] Sun, X. *et al.* PROTACs: Great opportunities for academia and industry. *Signal Transduct. Target Ther.* **4**, 64. 10.1038/s41392-019-0101-6 (2019). 31885879 10.1038/s41392-019-0101-6 PMC6927964

[42] Bekes, M., Langley, D. R. & Crews, C. M. PROTAC targeted protein degraders: The past is prologue. *Nat. Rev. Drug Discov.* **21**, 181–200. 10.1038/s41573-021-00371-6 (2022). 35042991 10.1038/s41573-021-00371-6 PMC8765495

[43] Gao, H., Sun, X. & Rao, Y. PROTAC technology: Opportunities and challenges. *ACS Med. Chem. Lett.* **11**, 237–240 (2020). 32184950 10.1021/acsmedchemlett.9b00597 PMC7073876

[44] Bond, A. G. *et al.* Development of BromoTag: A “Bump-and-Hole”–PROTAC system to induce potent, rapid, and selective degradation of tagged target proteins. *J. Med. Chem.* **64**, 15477–15502 (2021). 34652918 10.1021/acs.jmedchem.1c01532 PMC8558867

[45] Ran, F. A. *et al.* Double nicking by RNA-guided CRISPR Cas9 for enhanced genome editing specificity. *Cell* **154**, 1380–1389. 10.1016/j.cell.2013.08.021 (2013). 23992846 10.1016/j.cell.2013.08.021 PMC3856256

[46] Shen, B. *et al.* Efficient genome modification by CRISPR-Cas9 nickase with minimal off-target effects. *Nat. Methods* **11**, 399–402. 10.1038/nmeth.2857 (2014). 24584192 10.1038/nmeth.2857

[47] Morimoto, M., Nishida, T., Honda, R. & Yasuda, H. Modification of cullin-1 by ubiquitin-like protein Nedd8 enhances the activity of SCF(skp2) toward p27(kip1). *Biochem. Biophys. Res. Commun.* **270**, 1093–1096. 10.1006/bbrc.2000.2576 (2000). 10772955 10.1006/bbrc.2000.2576

[48] Baek, K. *et al.* NEDD8 nucleates a multivalent cullin-RING-UBE2D ubiquitin ligation assembly. *Nature* **578**, 461–466. 10.1038/s41586-020-2000-y (2020). 32051583 10.1038/s41586-020-2000-y PMC7050210

[49] Schultz, L. B., Chehab, N. H., Malikzay, A. & Halazonetis, T. D. p53 binding protein 1 (53BP1) is an early participant in the cellular response to DNA double-strand breaks. *J. Cell Biol.* **151**, 1381–1390 (2000). 11134068 10.1083/jcb.151.7.1381 PMC2150674

[50] Burma, S., Chen, B. P., Murphy, M., Kurimasa, A. & Chen, D. J. ATM phosphorylates histone H2AX in response to DNA double-strand breaks. *J. Biol. Chem.* **276**, 42462–42467 (2001). 11571274 10.1074/jbc.C100466200

[51] Smith, J., Tho, L. M., Xu, N. & Gillespie, D. A. The ATM–Chk2 and ATR–Chk1 pathways in DNA damage signaling and cancer. *Adv. Cancer Res.* **108**, 73–112 (2010). 21034966 10.1016/B978-0-12-380888-2.00003-0

[52] Lukas, C. *et al.* 53BP1 nuclear bodies form around DNA lesions generated by mitotic transmission of chromosomes under replication stress. *Nat. Cell Biol.* **13**, 243–253 (2011). 21317883 10.1038/ncb2201

[53] Mocali, A., Giovannelli, L., Dolara, P. & Paoletti, F. The comet assay approach to senescent human diploid fibroblasts identifies different phenotypes and clarifies relationships among nuclear size, DNA content, and DNA damage. *J. Gerontol. Ser. A: Biol. Sci. Med. Sci.* **60**, 695–701 (2005). 15983170 10.1093/gerona/60.6.695

[54] Dos Santos, Á. *et al.* DNA damage alters nuclear mechanics through chromatin reorganization. *Nucleic Acids Res.* **49**, 340–353 (2021). 33330932 10.1093/nar/gkaa1202 PMC7797048

[55] Zimmermann, A. *et al.* A new class of selective ATM inhibitors as combination partners of DNA double-strand break inducing cancer therapies. *Mol. Cancer Therap.* **21**, 859–870 (2022). 35405736 10.1158/1535-7163.MCT-21-0934 PMC9381122

[56] Kitagawa, R., Bakkenist, C. J., McKinnon, P. J. & Kastan, M. B. Phosphorylation of SMC1 is a critical downstream event in the ATM–NBS1–BRCA1 pathway. *Genes Dev.* **18**, 1423–1438 (2004). 15175241 10.1101/gad.1200304 PMC423193

[57] Smogorzewska, A. *et al.* Identification of the FANCI protein, a monoubiquitinated FANCD2 paralog required for DNA repair. *Cell* **129**, 289–301. 10.1016/j.cell.2007.03.009 (2007). 17412408 10.1016/j.cell.2007.03.009 PMC2175179

[58] Ferretti, S. *et al.* Discovery of WRN inhibitor HRO761 with synthetic lethality in MSI cancers. *Nature* **629**, 1–7 (2024). 10.1038/s41586-024-07350-y PMC11078746 38658754

[59] Zong, D. *et al.* Comprehensive mapping of cell fates in microsatellite unstable cancer cells supports dual targeting of WRN and ATR. *Genes Dev.* **37**, 913–928 (2023). 37932011 10.1101/gad.351085.123 PMC10691471

[60] Ohoka, N. *et al.* In vivo knockdown of pathogenic proteins via specific and nongenetic inhibitor of apoptosis protein (IAP)-dependent protein erasers (SNIPERs). *J. Biol. Chem.* **292**, 4556–4570. 10.1074/jbc.M116.768853 (2017). 28154167 10.1074/jbc.M116.768853 PMC5377772

[61] Raina, K. *et al.* PROTAC-induced BET protein degradation as a therapy for castration-resistant prostate cancer. *Proc. Natl. Acad. Sci. USA* **113**, 7124–7129. 10.1073/pnas.1521738113 (2016). 27274052 10.1073/pnas.1521738113 PMC4932933

[62] Zhou, B. *et al.* Discovery of a small-molecule degrader of bromodomain and extra-terminal (BET) proteins with picomolar cellular potencies and capable of achieving tumor regression. *J. Med. Chem.* **61**, 462–481. 10.1021/acs.jmedchem.6b01816 (2018). 28339196 10.1021/acs.jmedchem.6b01816 PMC5788414

[63] Saenz, D. T. *et al.* Novel BET protein proteolysis-targeting chimera exerts superior lethal activity than bromodomain inhibitor (BETi) against post-myeloproliferative neoplasm secondary (s) AML cells. *Leukemia* **31**, 1951–1961. 10.1038/leu.2016.393 (2017). 28042144 10.1038/leu.2016.393 PMC5537055

[64] Burslem, G. M. *et al.* The advantages of targeted protein degradation over inhibition: An RTK case study. *Cell Chem. Biol.* **25**, 67-77.e63. 10.1016/j.chembiol.2017.09.009 (2018). 29129716 10.1016/j.chembiol.2017.09.009 PMC5831399

[65] Dias, M. P., Moser, S. C., Ganesan, S. & Jonkers, J. Understanding and overcoming resistance to PARP inhibitors in cancer therapy. *Nat. Rev. Clin. Oncol.* **18**, 773–791. 10.1038/s41571-021-00532-x (2021). 34285417 10.1038/s41571-021-00532-x

[66] Noordermeer, S. M. *et al.* The shieldin complex mediates 53BP1-dependent DNA repair. *Nature* **560**, 117–121 (2018). 30022168 10.1038/s41586-018-0340-7 PMC6141009

