# Supplementary material for: PROTAC-mediated conditional degradation of the WRN helicase as a potential strategy for selective killing of cancer cells with microsatellite instability
Source: Sci Rep. 2024 Sep 6;14:20824. doi: 10.1038/s41598-024-71160-5 (PMC11379953; doi:10.1038/s41598-024-71160-5)
Supplement: Supplementary file 1 — Supplementary Information 1. [file 41598_2024_71160_MOESM1_ESM.docx]

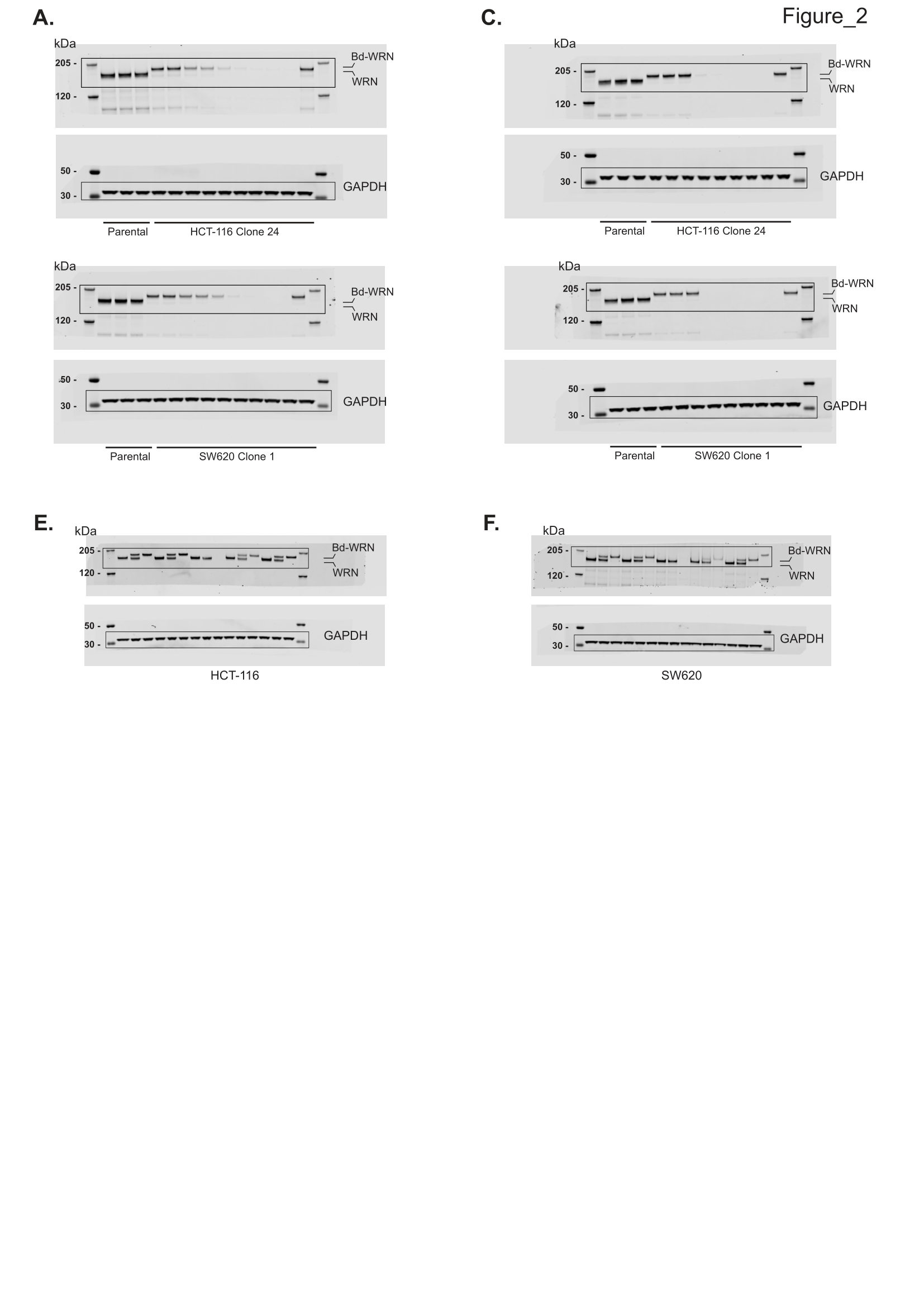


**Uncropped blots showing rapid, PROTAC-inducible and proteasome dependent WRN degradation in MSI HCT-116 clone 24 and MSS SW620 clone 1 from Figure 2.**


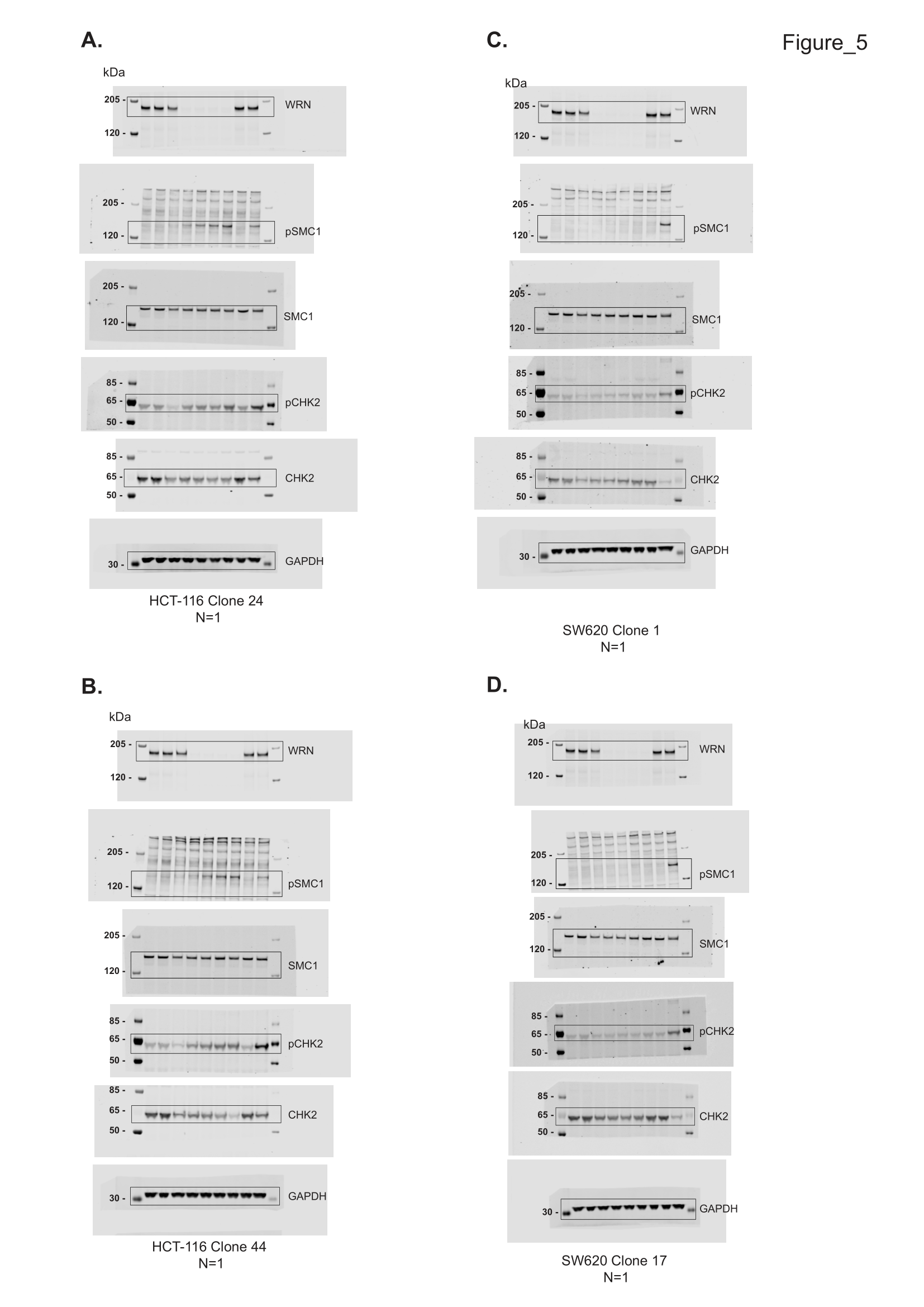


**Uncropped blots showing WRN degradation in MSI but not MSS, cells causes checkpoint activation from Figure 5.**


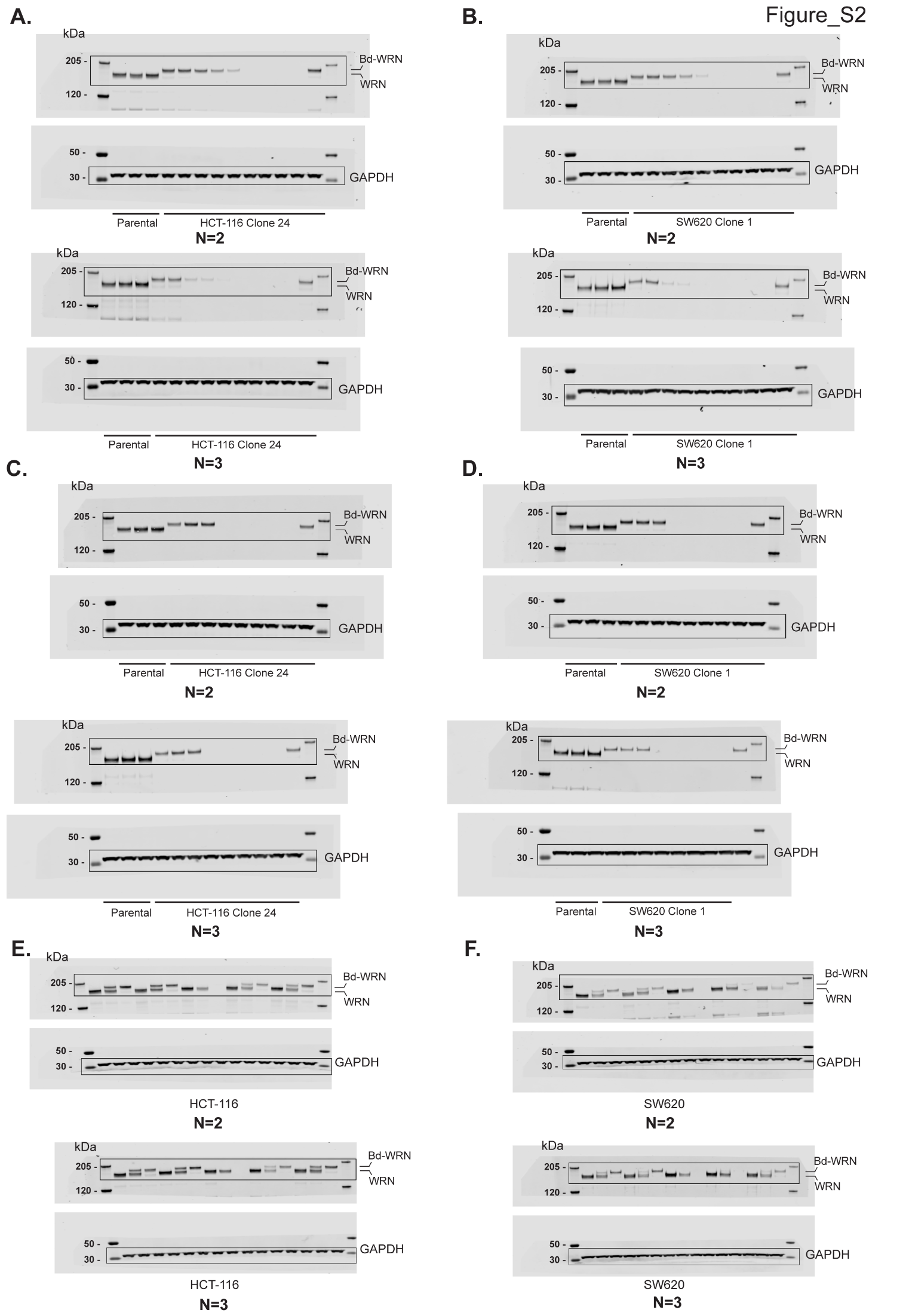


**Uncropped blots showing rapid, PROTAC-inducible and proteasome dependent WRN degradation in MSI HCT-116 clone 24 and MSS SW620 clone 1 from Figure S2.**


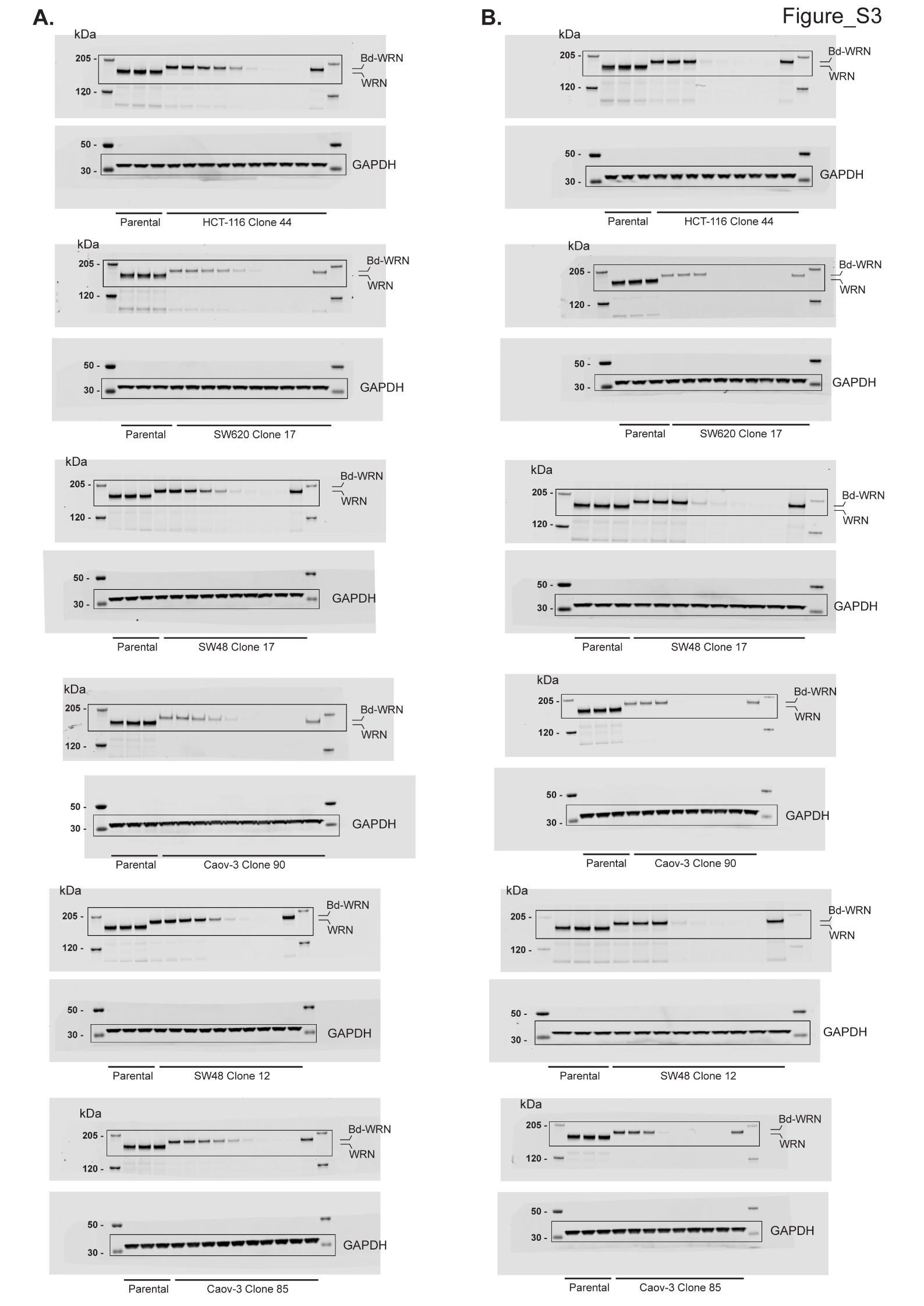


**Uncropped blots showing rapid, PROTAC-inducible WRN degradation in MSS SW620 and Caov-3 cells and MSI HCT-116 and SW48 cells from Figure S3.**


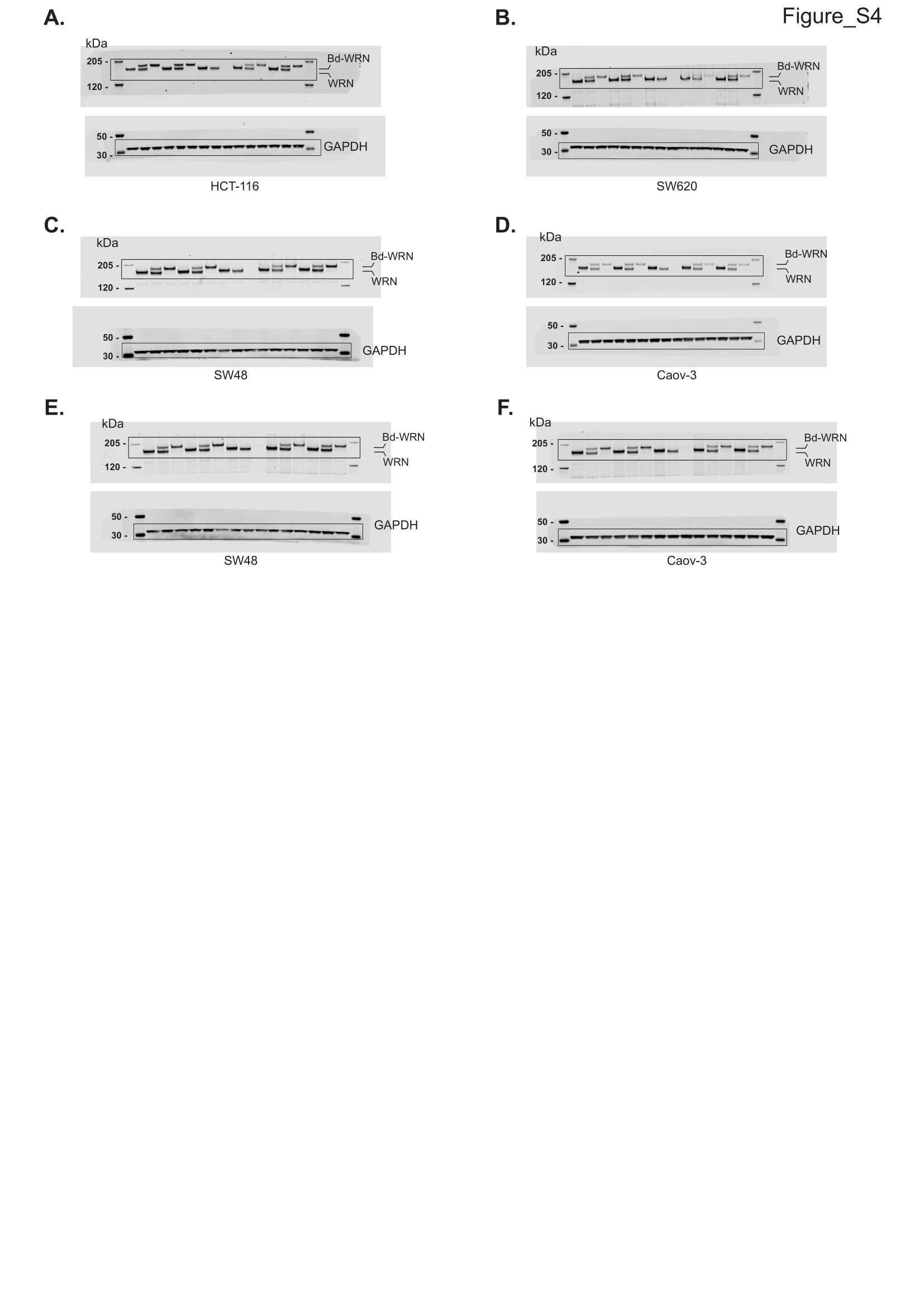


**Uncropped blots showing PROTAC-inducible WRN degradation in MSS and MSI cells is proteasome dependent from Figure S4.**


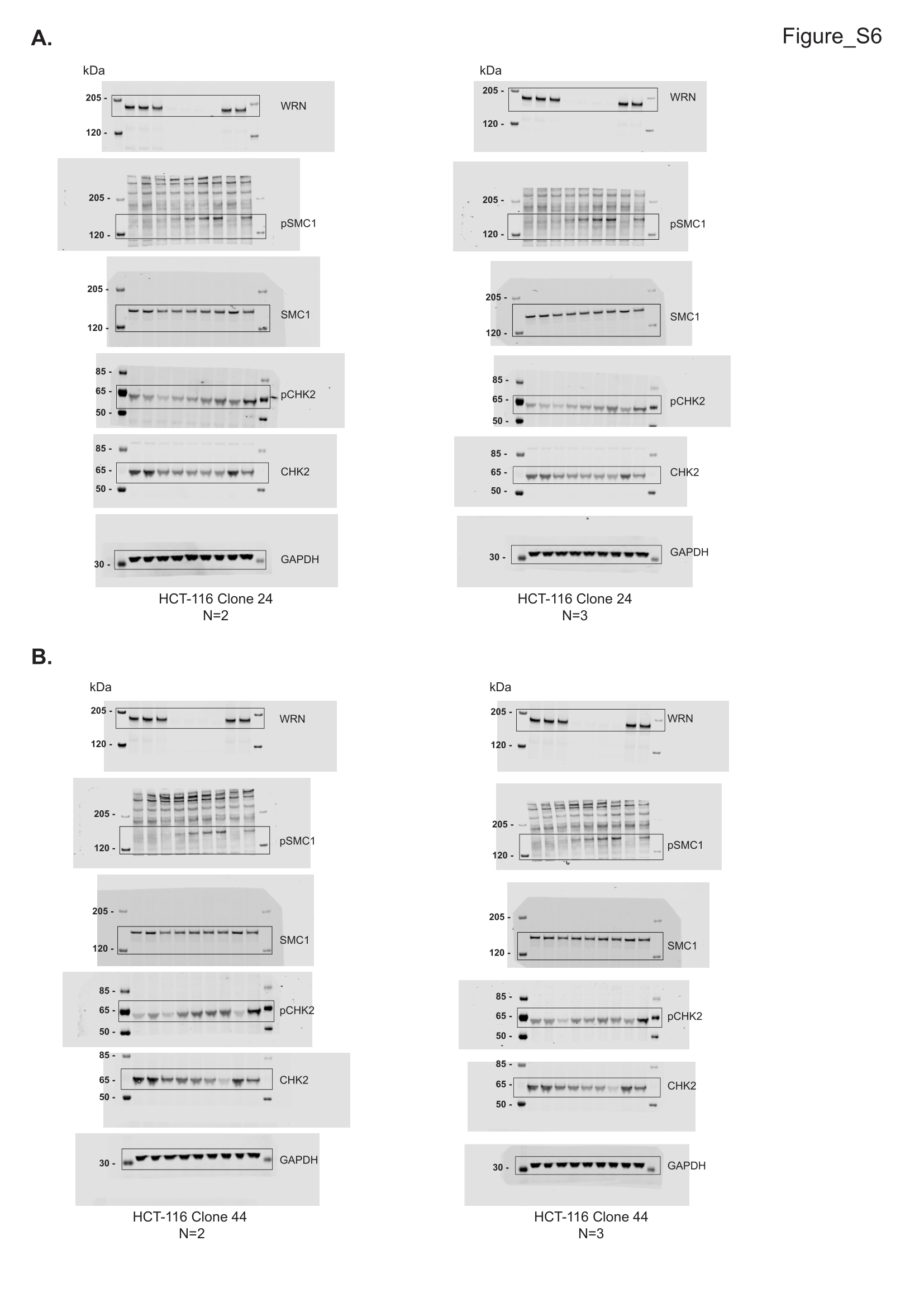


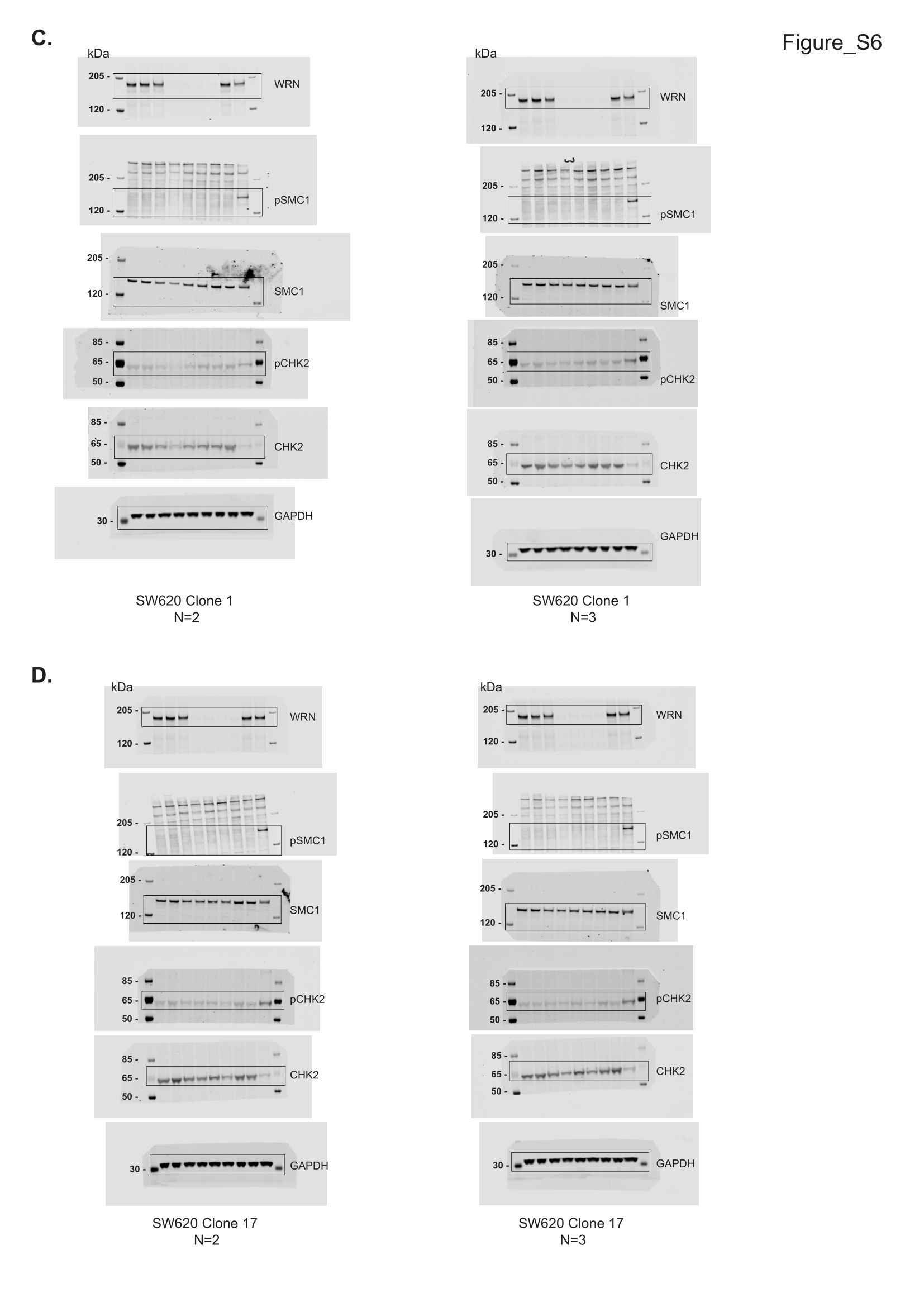


**Uncropped blots showing WRN degradation in MSI but not MSS, cells causes checkpoint activation from Figure S6.**


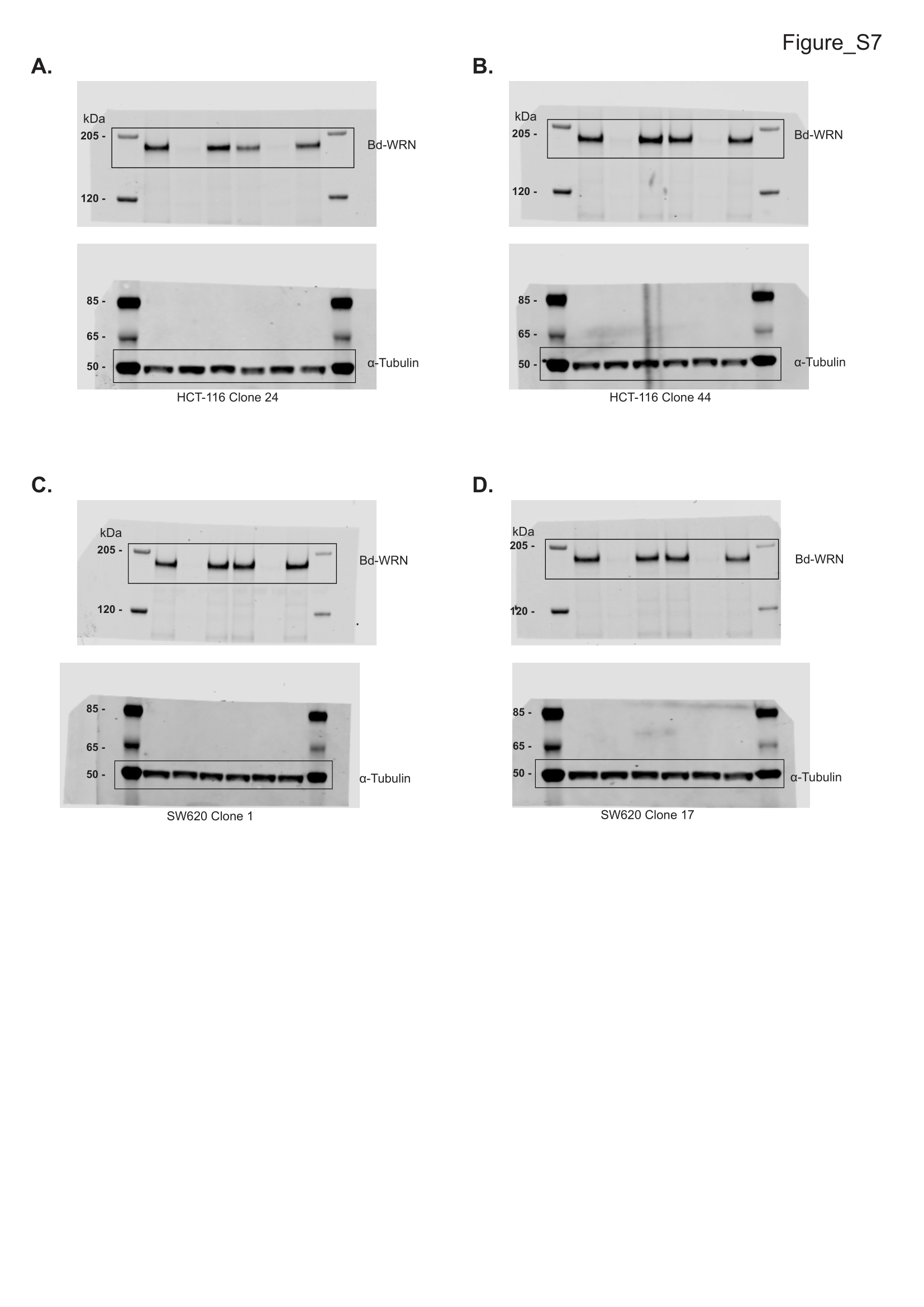


**Uncropped blots showing AGB-1-dependent degradation of Bd-WRN in mCherry HCT‑116 and BFP SW620 clones from Figure S7.**
