# Supplementary material for: PROTAC-mediated conditional degradation of the WRN helicase as a potential strategy for selective killing of cancer cells with microsatellite instability
Source: Sci Rep. 2024 Sep 6;14:20824. doi: 10.1038/s41598-024-71160-5 (PMC11379953; doi:10.1038/s41598-024-71160-5)
Supplement: Supplementary file 2 — Supplementary Information 2. [file 41598_2024_71160_MOESM2_ESM.docx]

**Supplementary Information**

**­PROTAC-mediated conditional degradation of the WRN helicase as a potential strategy for selective killing of cancer cells with microsatellite instability**

Vikram Tejwani^1^, Thomas Carroll^1^, Thomas Macartney^1^, Susanne Bandau^2^, Constance Alabert^2^, Giulia Saredi^1^, Rachel Toth^1^ & John Rouse^1^

^1^MRC Protein Phosphorylation and Ubiquitylation Unit, ^2^Division of Molecular, Cell and Developmental Biology, School of Life Sciences, Wellcome Trust Biocentre, University of Dundee, DD1 5EH, UK.

**Supplementary Information Inventory**

**Figure S1. Junction PCR and Genotypic analysis of BromoTag WRN clones.**

**Figure S2. Rapid, PROTAC-inducible and proteasome dependent WRN degradation in MSI HCT-116 clone 24 and MSS SW620 clone 1.**

**Figure S3. Rapid, PROTAC-inducible WRN degradation in MSS SW620 and Caov-3 cells and MSI HCT-116 and SW48 cells.**

**Figure S4. PROTAC-inducible WRN degradation in MSS and MSI cells is proteasome dependent.**

**Figure S5. WRN degradation in MSI but not MSS, cells causes DNA breaks and enlarged nuclei.**

**Figure S6. WRN degradation in MSI but not MSS, cells causes checkpoint activation.**

**Figure S7. AGB-1 induces degradation of Bd-WRN in mCherry HCT-116 and BFP SW620 clones.**

**Figure S8. Flow cytometry plots of MCA assays.**


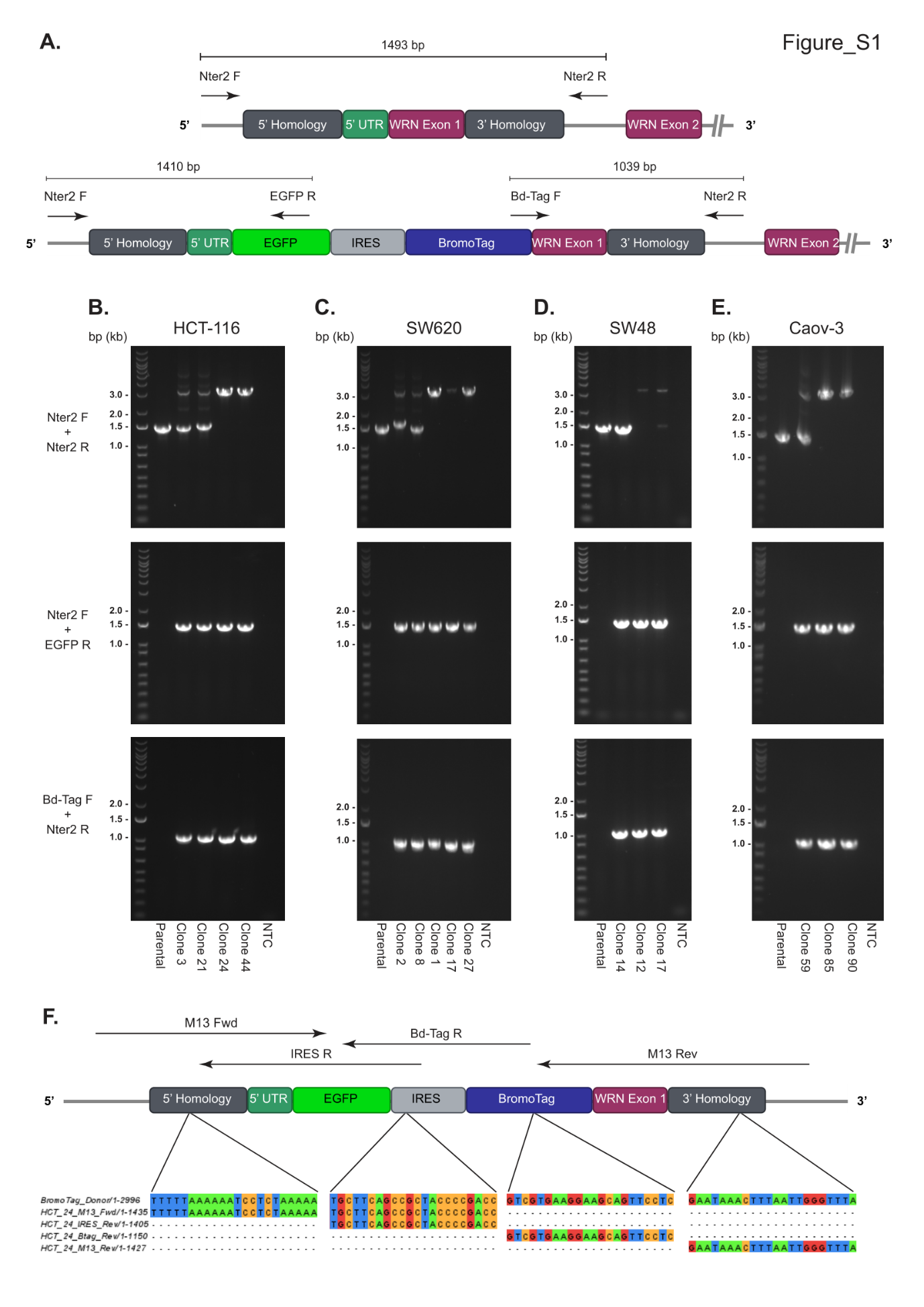


**Figure S1. Junction PCR and Genotypic analysis of BromoTag WRN clones. (A)** Schematic representation of the junction PCR analysis strategy for parental (top) and Bd-WRN KI clones (bottom). All cells were amplified by three primer pairs: the Nter2 F and R (flanking the KI insertion site, outside homology regions), the Nter2 F and EGFP R, and the Bd-Tag F and Nter2 R. **(B)** 1.0% w/v DNA agarose gels of products from the junction PCR analysis strategy described in **(A)** for HCT-116 parental and knock-in (KI) clones. The top, middle and bottom panels correspond to products from the Nter2 F + R, and Nter2 F + EGFP R and Bd-Tag F + Nter2 R reactions, respectively. NTC stands for ‘No template control’. **(C)** Same as in **(B)** for SW620 parental and KI clones. **(D)** Same as in **(B)** for SW48 parental and KI­­­­ clones. **(E)** Same as in **(B)** for Caov-3 parental and KI clones. **(F)** Schematic representation of the StrataClone genotyping strategy. Sequence alignments from a single StrataClone colony for HCT-116 clone 24 are shown highlighting the in-frame knock-in of the BromoTag construct. Primers used were M13 Fwd, M13 Rev, Bd-Tag Rev and the IRES Rev.­­


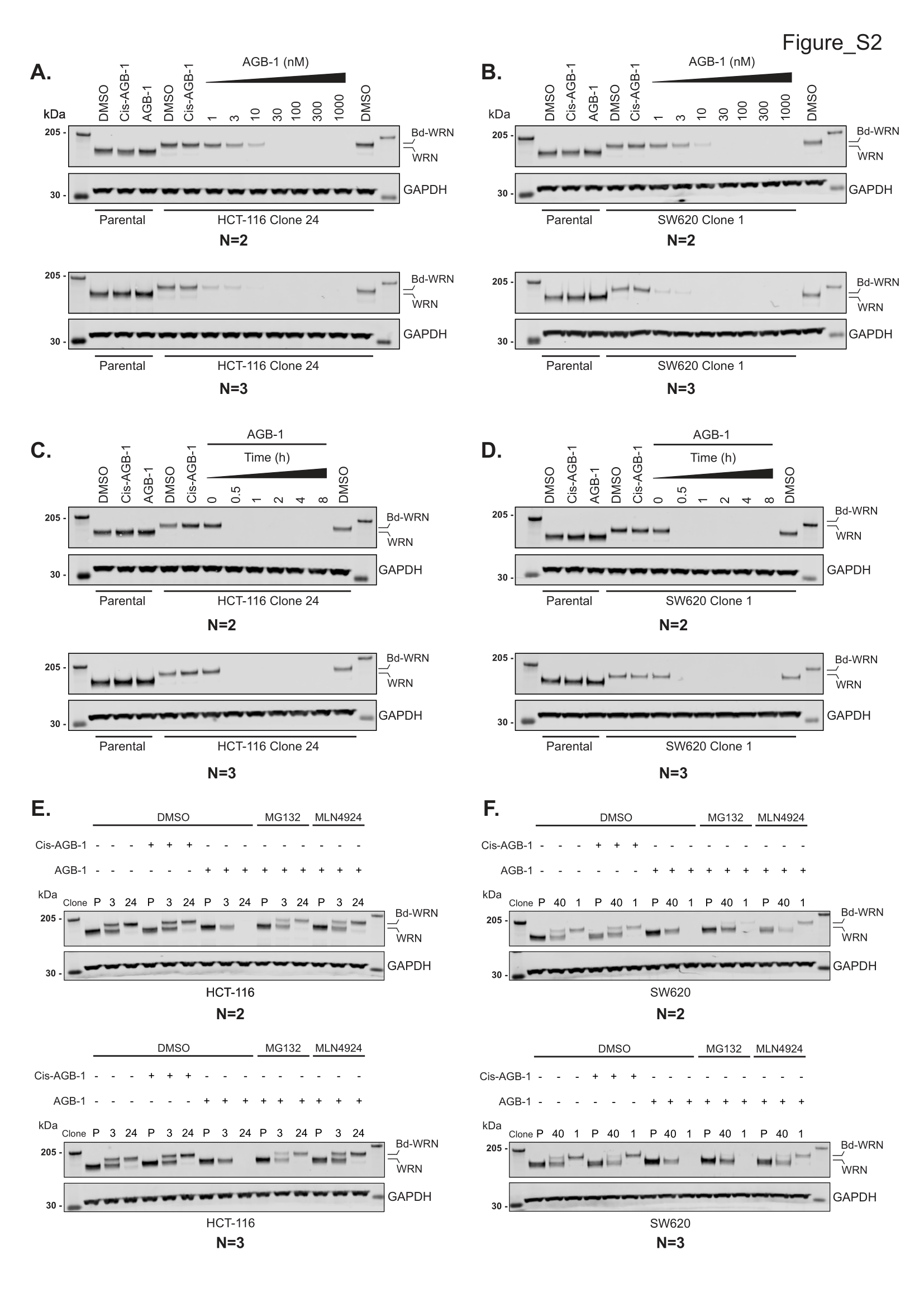


**Figure S2. Rapid, PROTAC-inducible and proteasome dependent WRN degradation in MSI HCT-116 clone 24 and MSS SW620 clone 1. (A)** Second (top) and third (bottom) biological repeats for HCT-116 clone 24 shown in Figure 2A. **(B)** Second (top) and third (bottom) biological repeats for SW620 clone 1 shown in Figure 2A. **(C)** Second (top) and third (bottom) biological repeats for HCT-116 clone 24 shown in Figure 2C. **(D)** Second (top) and third (bottom) biological repeats for SW620 clone 1 shown in Figure 2C. **(E)** Second (top) and third (bottom) biological repeats for HCT-116 clone 24 shown in Figure 2E. **(F)** Second (top) and third (bottom) biological repeats for SW620 clone 1 shown in Figure 2F.


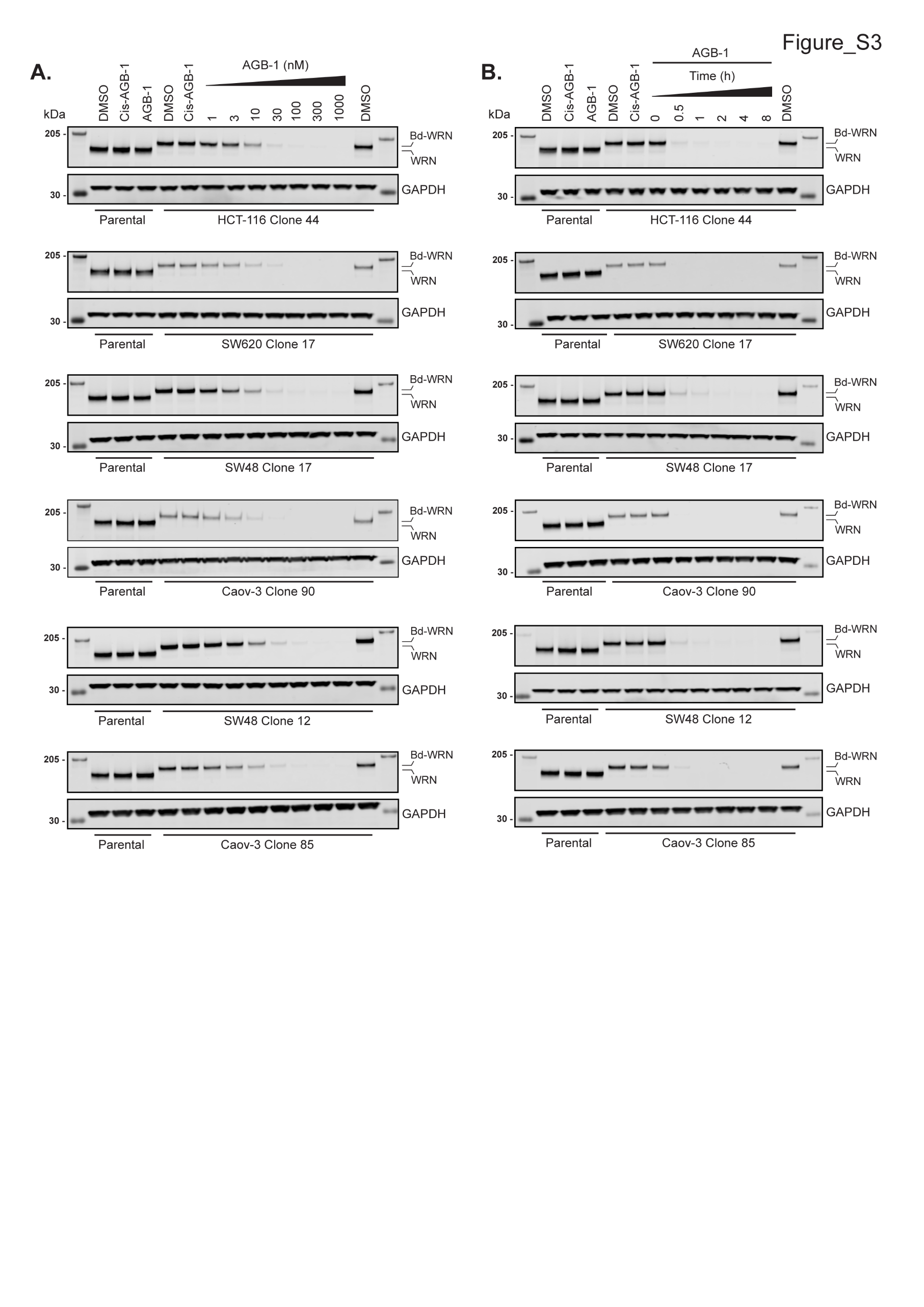


**Figure S3. Rapid, PROTAC-inducible WRN degradation in MSS SW620 and Caov-3 cells and MSI HCT-116 and SW48 cells. (A)** Representative SDS-PAGE and western blot analysis of lysates from HCT-116 clone 44, SW620 clone 17, SW48 clones 12 and 17 and Caov-3 clones 85 and 90 showing degradation of Bd-WRN with increasing concentrations of AGB-1, after 3h of treatment. 3h Cis-AGB-1 (1 µM) and DMSO (0.1%) were used as controls. Western blots shown are from a single biological repeat. **(B)** Western blot analysis showing the time-dependent degradation of Bd-WRN in the same clones as in **(A)**, at a fixed AGB-1 concentration (0.3 µM). Parental cells were also included for each cell type. 8h cis-AGB-1 (1 µM) and DMSO (0.1%) treatments were used as controls. Western blots shown are from a single biological repeat.


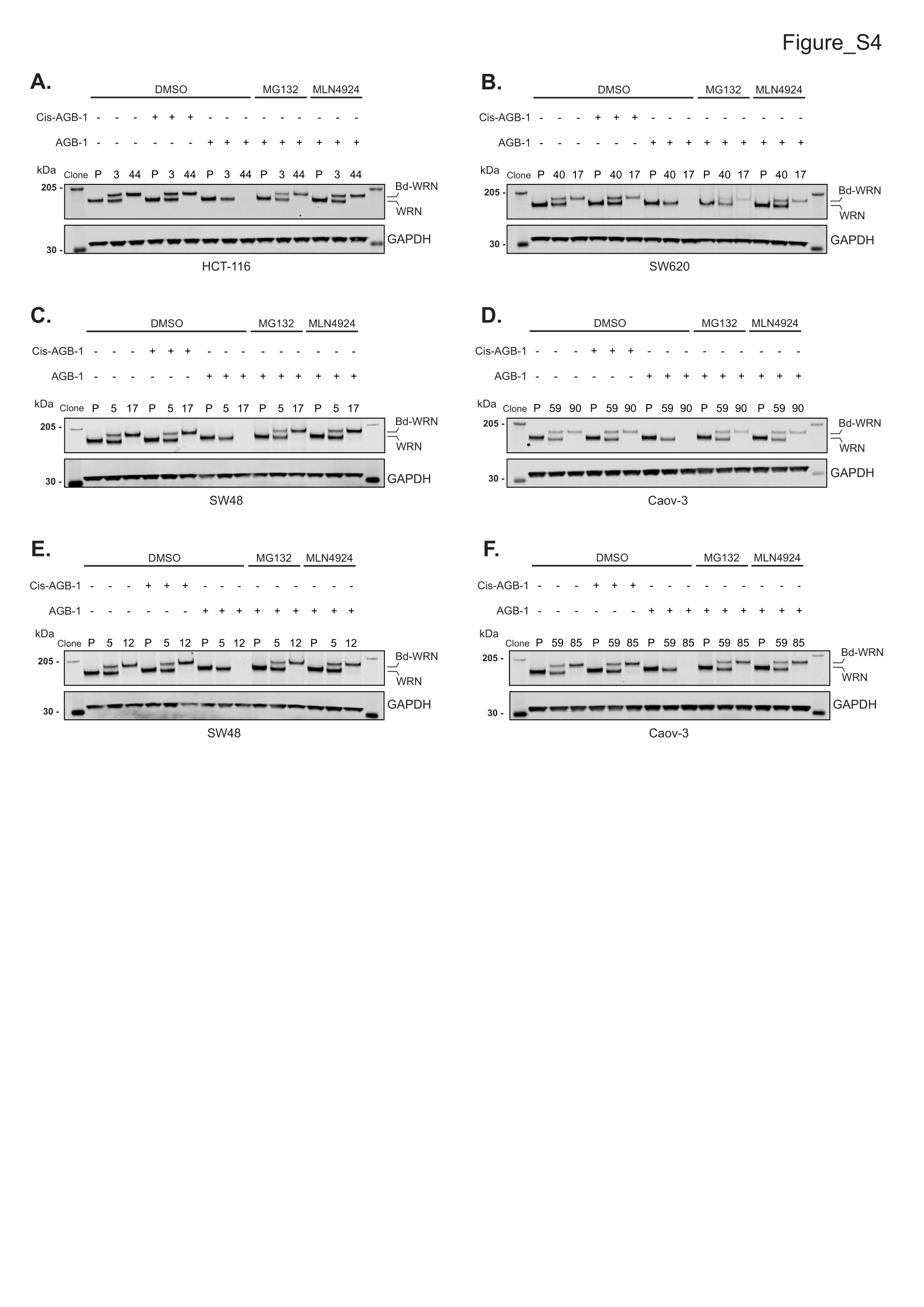


**Figure S4. PROTAC-inducible WRN degradation in MSS and MSI cells is proteasome dependent. (A)** Western blot analysis showing that AGB-1 mediated degradation of Bd-WRN is dependent on the proteasome in HCT-116 parental cells (P), clone 3 (untagged and Bd-tagged WRN) and clone 44 (Bd-tagged WRN only). Cells were preincubated for 1h with the 26S proteasome inhibitor MG132 (50 µM) or the NEDDylation inhibitor MLN4924 (3 µM) or DMSO (0.1%) before treatment with 0.3 µM AGB-1 or cis-AGB-1 for a further 3h. **(B)** The same as in **(A)** done with SW620 parental cells (P), clone 40 (untagged and Bd-tagged WRN) and clone 17 (Bd-tagged WRN only). **(C)** The same as in **(A)** done with SW48 parental cells (P), clone 5 (untagged and Bd-tagged WRN) and clone 17 (Bd-tagged WRN only). **(D)** The same as in **(A)** done with Caov-3 parental cells (P), clone 59 (untagged and Bd-tagged WRN) and clone 90 (Bd-tagged WRN only). **(E)** The same as in **(A)** done with SW48 parental cells (P), clone 5 (untagged and Bd-tagged WRN) and clone 12 (Bd-tagged WRN only). **(F)** The same as in **(A)** done with Caov-3 parental cells (P), clone 59 (untagged and Bd-tagged WRN) and clone 85 (Bd-tagged WRN only).


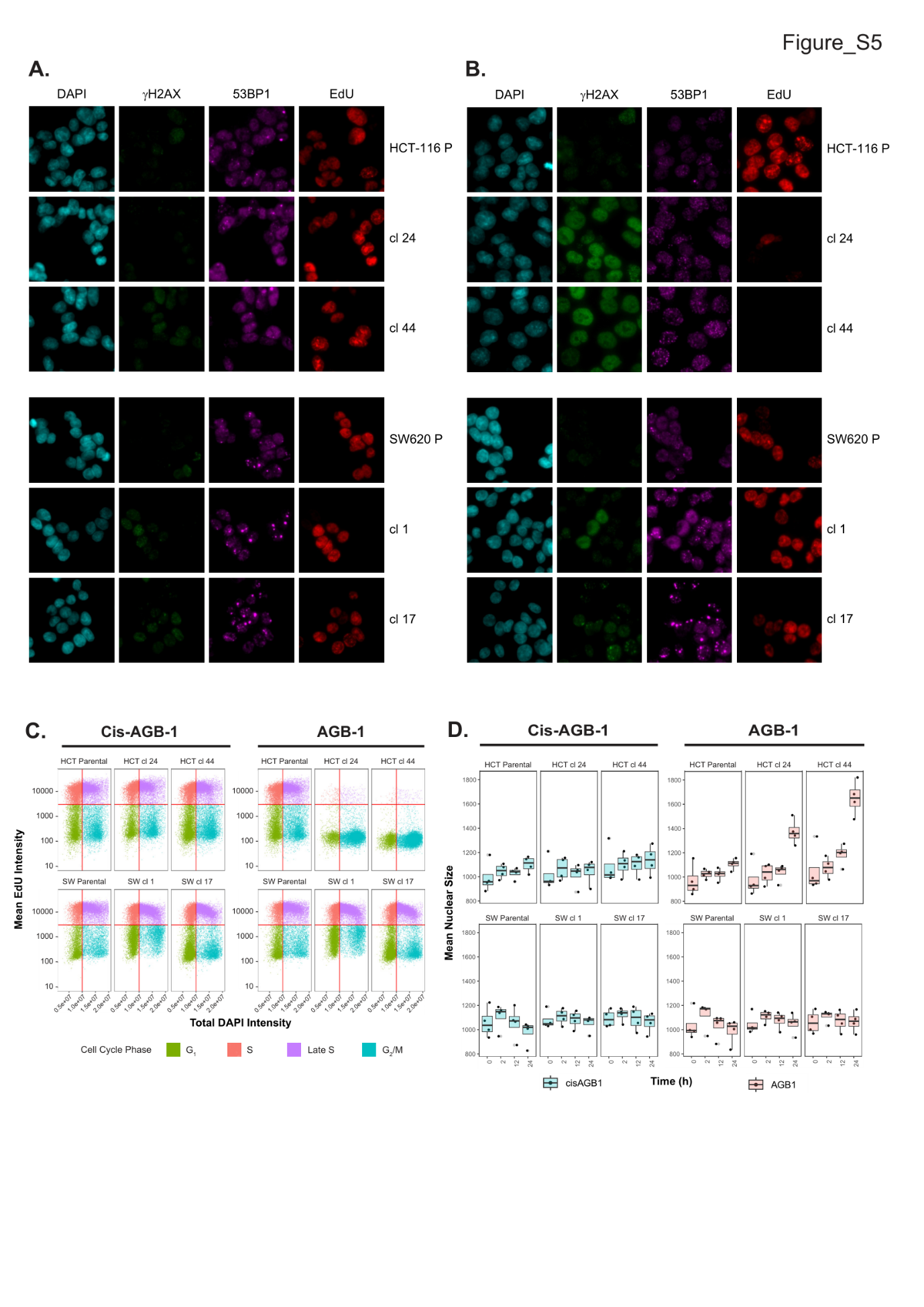


**Figure S5. WRN degradation in MSI but not MSS, cells causes DNA breaks and enlarged nuclei. (A)**, **(B)** Representative images from one of four biological repeats (*n=4*) used to quantify γH2AX intensities and 53BP1 foci in Figure 4 either 0 hours **(A)** or 24 hours **(B)** after treatment with 0.3 µM AGB-1. **(C)** Cell cycle analysis from Figure 4F coloured to represent thresholding of cells in G_1_ (green), early S (red), late S (purple) and G_2_/M (cyan). **(D)** Quantification of mean nuclear size of G_2_/M cells after 24 hours of treatment with 0.3 µM Cis-AGB-1 or AGB-1 from all four biological repeats (*n=4)*. Mean nuclear size of G_2_/M cells from each biological repeat is represented by an individual point.


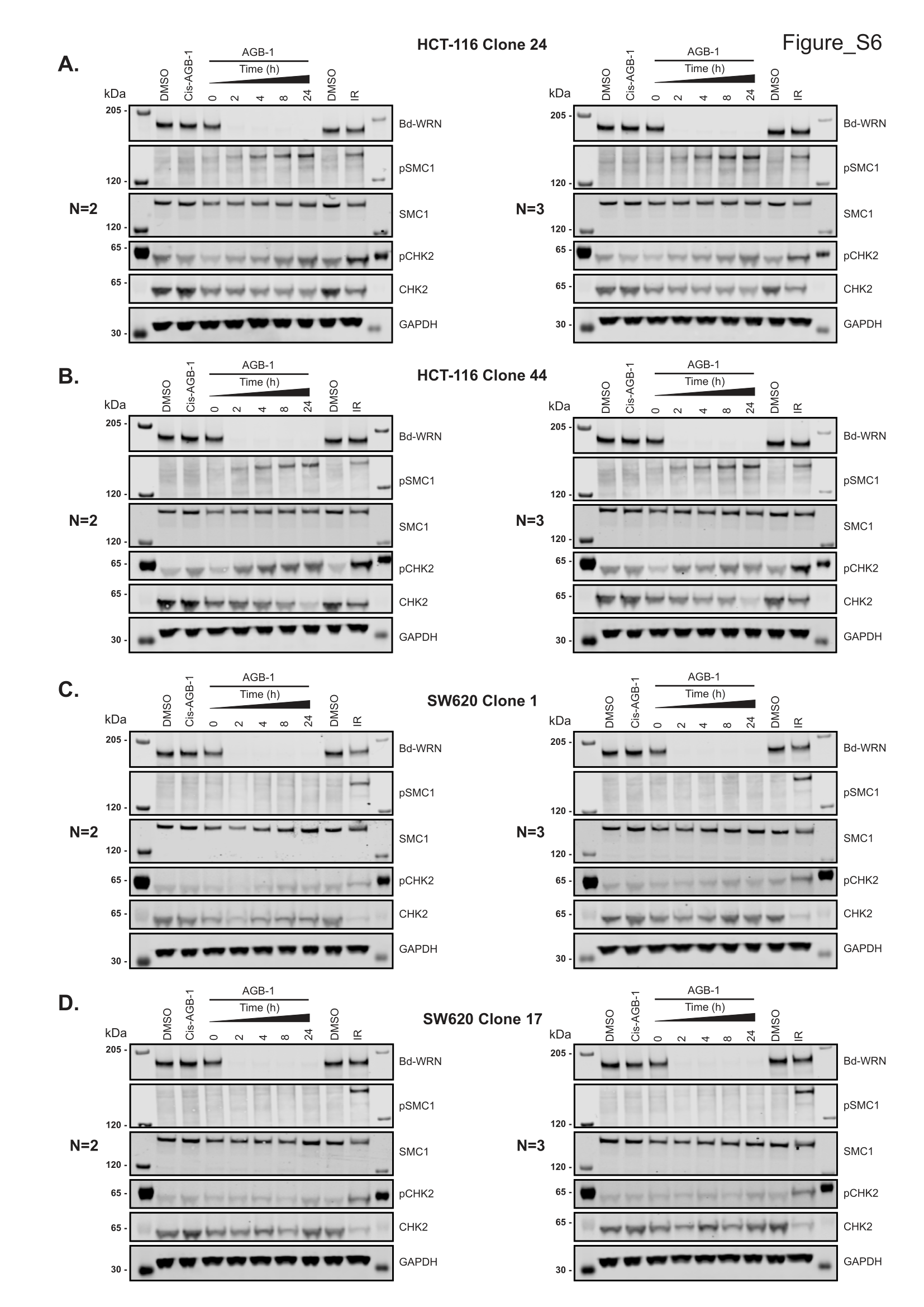


**Figure S6. WRN degradation in MSI but not MSS, cells causes checkpoint activation. (A)** Second (left) and third (right) biological repeats for HCT-116 clone 24 shown in Figure 5A. **(B)** Second (left) and third (right) biological repeats for HCT-116 clone 44 shown in Figure 5B. **(C)** Second (left) and third (right) biological repeats for SW620 clone 1 shown in Figure 5C. **(D)** Second (left) and third (right) biological repeats for SW620 clone 17 shown in Figure 5D.


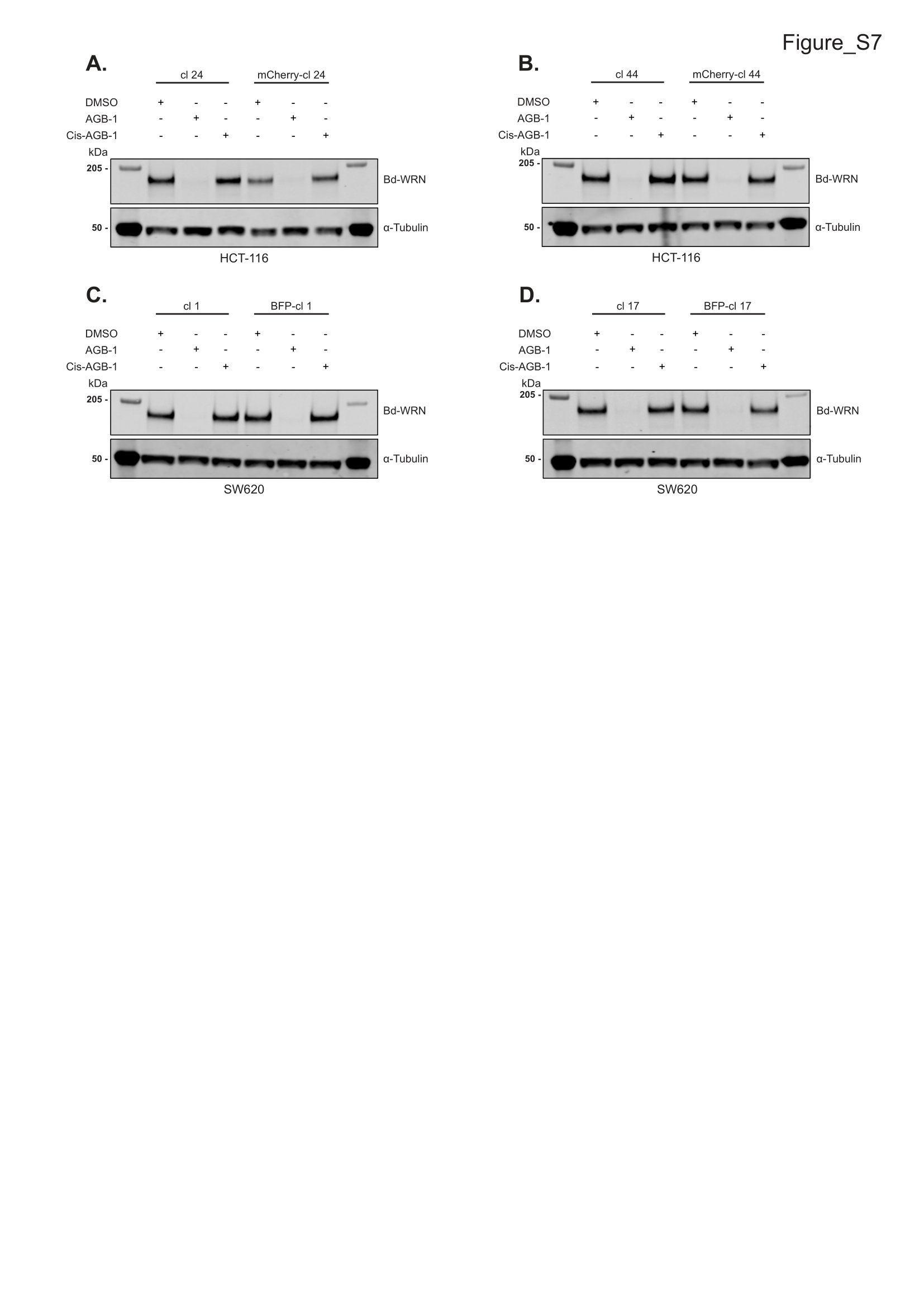


**Figure S7. AGB-1 induces degradation of Bd-WRN in mCherry HCT-116 and BFP SW620 clones. (A)** Western blot analysis of lysates from HCT-116 Bd-WRN clone 24 and mCherry Bd-WRN clone 24 showing degradation of Bd-WRN with 0.3 µM AGB-1 for 3h. 3h Cis-AGB-1 (0.3 µM) and DMSO (0.1%) treatments were used as controls. **(B)** The same as in **(A)**, with HCT-116 Bd-WRN clone 44 and mCherry Bd-WRN clone 44. **(C)** The same as in **(A)**, with SW620 Bd-WRN clone 1 and BFP Bd-WRN clone 1. **(D)** The same as in **(A)**, with SW620 Bd-WRN clone 17 and BFP Bd-WRN clone 17. All western blots shown are from a single biological repeat.


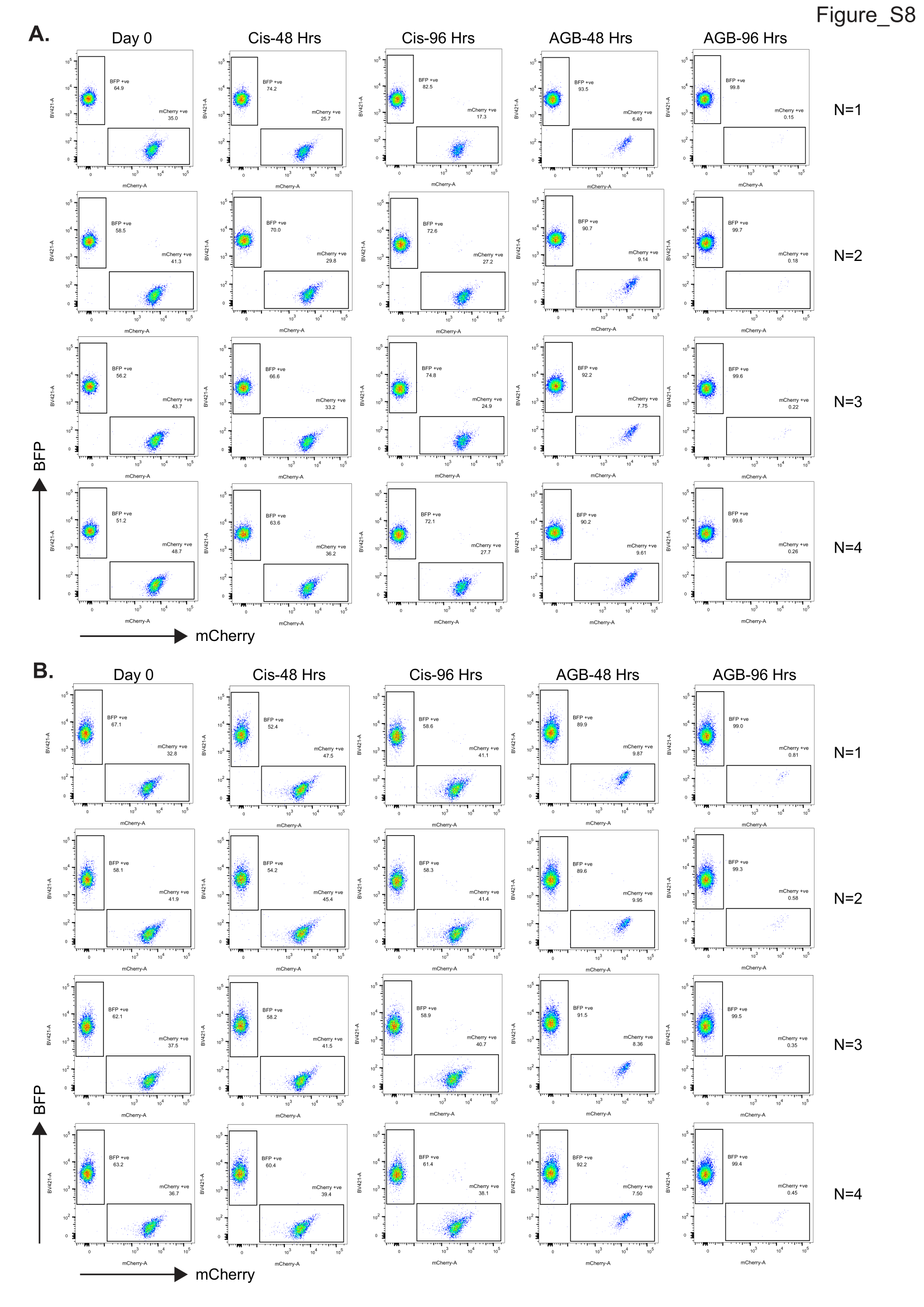


**Figure S8. Flow cytometry plots of MCA assays.** **(A)** Flow cytometry dot plots from all four biological repeats (*n=4*) of the MCA with mCherry HCT-116 Bd-WRN clone 24 and BFP SW620 Bd-WRN clone 1 summarised in Figure 6B. **(B)** Flow cytometry dot plots from all four biological repeats (*n=4*) of the MCA with mCherry HCT-116 Bd-WRN clone 44 and BFP SW620 Bd-WRN clone 17 summarised in Figure 6C.
